# Improved mitochondrial function in the hearts of sarcolipin-deficient dystrophin and utrophin double-knockout mice

**DOI:** 10.1172/jci.insight.170185

**Published:** 2024-04-02

**Authors:** Satvik Mareedu, Nadezhda Fefelova, Cristi L. Galindo, Goutham Prakash, Risa Mukai, Junichi Sadoshima, Lai-Hua Xie, Gopal J. Babu

**Affiliations:** 1 Department of Cell Biology and Molecular Medicine, New Jersey Medical School, Rutgers University, Newark, New Jersey, USA.; 2 Vascular Medicine Institute and Division of Cardiology, Department of Medicine, University of Pittsburgh, Pittsburgh, Pennsylvania, USA.

**Keywords:** Metabolism, Calcium, Mitochondria

## Abstract

Duchenne muscular dystrophy (DMD) is a progressive muscle-wasting disease associated with cardiomyopathy. DMD cardiomyopathy is characterized by abnormal intracellular Ca^2+^ homeostasis and mitochondrial dysfunction. We used dystrophin and utrophin double-knockout (*mdx:utrn^–/–^*) mice in a sarcolipin (SLN) heterozygous-knockout (*sln^+/–^*) background to examine the effect of SLN reduction on mitochondrial function in the dystrophic myocardium. Germline reduction of SLN expression in *mdx:utrn^–/–^* mice improved cardiac sarco/endoplasmic reticulum (SR) Ca^2+^ cycling, reduced cardiac fibrosis, and improved cardiac function. At the cellular level, reducing SLN expression prevented mitochondrial Ca^2+^ overload, reduced mitochondrial membrane potential loss, and improved mitochondrial function. Transmission electron microscopy of myocardial tissues and proteomic analysis of mitochondria-associated membranes showed that reducing SLN expression improved mitochondrial structure and SR-mitochondria interactions in dystrophic cardiomyocytes. These findings indicate that SLN upregulation plays a substantial role in the pathogenesis of cardiomyopathy and that reducing SLN expression has clinical implications in the treatment of DMD cardiomyopathy.

## Abstract 2

In dystrophic hearts, reducing sarcolipin expression improved sarco/endoplasmic reticulum calcium handling, prevents mitochondrial calcium overload and improved mitochondrial structure and function.

## Graphical abstract

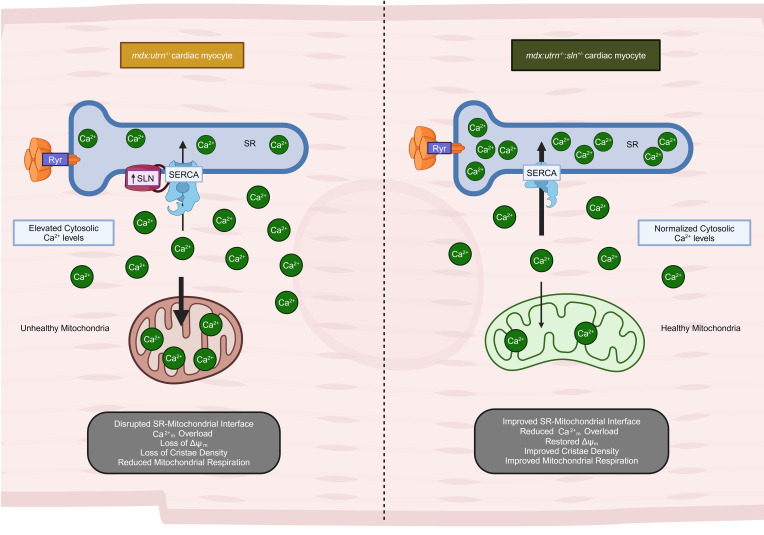

## Introduction

Duchenne muscular dystrophy (DMD) is an X-linked disorder caused by mutations in the dystrophin gene. Lack of dystrophin, being the prime cause of the disease, displays clinical manifestations, such as continuous reduction in muscle function, making children bedridden by the start of the second decade. As age progresses, patients with DMD start to manifest altered cardiac parameters and respiratory weakness (1, 2). While progressive respiratory failure remains the most common cause of death, increased life expectancy has revealed the importance of cardiomyopathy, which accounts for approximately 40% of deaths in the DMD population (3). In this context, it is important to understand the underlying molecular mechanisms causing dystrophic cardiomyopathy and heart failure to identify novel therapeutic targets.

In DMD, abnormal intracellular calcium (Ca^2+^_i_) homeostasis is considered one of the main secondary changes that cause muscle pathology and cardiomyopathy (4). The absence of dystrophin protein destabilizes the membrane integrity, resulting in stress-induced membrane tears, and is associated with Ca^2+^ leak, leading to abnormal elevation of Ca^2+^_i_ concentration, upregulation of inflammatory factors, and mitochondrial dysfunction, ultimately leading to muscle degeneration, necrosis, and cardiomyopathy (4–8). In addition to the activation of various sarcolemmal Ca^2+^ channels, defects in sarco/endoplasmic reticulum (SR) Ca^2+^ handling are shown to contribute to the chronic elevation of Ca^2+^_i_ concentration (4). Under normal physiological conditions, increased cytoplasmic Ca^2+^ levels have been shown to increase mitochondrial Ca^2+^ (Ca^2+^_m_) uptake, resulting in increased metabolism (9). On the other hand, during pathological conditions, abnormal elevation of Ca^2+^_m_ content can result in the opening of the mitochondrial permeability transition pore, causing mitochondrial dysfunction and cell death (10, 11). It has been shown that in dystrophic hearts, Ca^2+^_m_ levels are elevated and associated with defective mitophagy (12, 13). During cardiac pathology, SR-mediated cytoplasmic Ca^2+^ rise caused by increased SR Ca^2+^ leak via ryanodine receptor (RyR), along with decreased SR Ca^2+^ uptake via sarco/endoplasmic reticulum Ca^2+^ ATPase (SERCA), can increase Ca^2+^_m_ concentration, indicating that there is a close association between SR and mitochondria (14, 15). Therefore, it is important to determine whether improving SR Ca^2+^ handling can improve Ca^2+^_m_ handling and mitochondrial function in dystrophic hearts.

We recently demonstrated that the expression of sarcolipin (SLN), a potent inhibitor of the SERCA pump, is increased in dystrophic muscles and hearts of animal models and human DMD (16, 17). Furthermore, in mouse models of DMD, reducing SLN expression is sufficient to improve SR Ca^2+^ handling and mitigate DMD and associated cardiomyopathy (17, 18). Our studies also demonstrated that reducing SLN expression can improve mitochondria mediated energy metabolism in *mdx* muscles (19). However, the causal link between SLN upregulation, SR Ca^2+^ handling, and Ca^2+^_m_ load and their effect on mitochondrial function in dystrophic myocardium has not been studied to our knowledge. Therefore, the major goal of this study was to establish whether SLN upregulation is linked to mitochondrial dysfunction in dystrophic myocardium. Here, using dystrophin and utrophin double-knockout (*mdx:utrn^–/–^*) mice as a model system, we tested the hypothesis that reducing SLN expression is sufficient to improve cardiac SERCA function, SR Ca^2+^ cycling and thereby Ca^2+^_m_ content, and mitochondrial function in dystrophic myocardium.

## Results

### Reducing SLN expression improves SR Ca^2+^ handling and cardiac contractility in mdx:utrn^–/–^ mice.

We previously demonstrated that ablating 1 SLN allele (*sln^+/–^*) is sufficient to normalize SLN expression, mitigate severe muscular dystrophy, and improve cardiac function in *mdx:utrn^–/–^* mice (17). Therefore, to test our hypothesis, we chose *mdx:utrn^–/–^:sln^+/–^* mice.

We first measured cardiac function using M-mode echocardiography. Results showed that the systolic function of the heart, as measured by the left ventricular (LV) ejection fraction (EF) and fractional shortening (FS), was significantly improved in *mdx:utrn^–/–^:sln^+/–^* mice compared with that of age-and sex-matched *mdx:utrn^–/–^* mice (Supplemental Table 1; supplemental material available online with this article; https://doi.org/10.1172/jci.insight.170185DS1). Pulse wave Doppler analysis showed that the ratio between early (E) and late (atrial-A) ventricular filling velocity (E/A) was significantly reduced, and the myocardial performance index (MPI) was significantly increased in *mdx:utrn^–/–^* mice compared with that of WT controls (Supplemental Figure 1). On the other hand, the E/A ratio was significantly increased, and MPI was decreased, in *mdx:utrn^–/–^:sln^+/–^* mice (Supplemental Figure 1). Histopathological examination of ventricular sections using Picrosirius red (PSR) staining showed that *mdx:utrn^–/–^* mice developed severe cardiac fibrosis as evidenced by increased collagen accumulation. On the other hand, collagen accumulation was significantly reduced in the ventricles of *mdx:utrn^–/–^:sln^+/–^* mice (Supplemental Figure 2). These findings together indicate that reducing SLN expression attenuated cardiac fibrosis, prevented diastolic dysfunction, and improved the overall cardiac function in *mdx:utrn^–/–^* mice.

**Figure 1:**
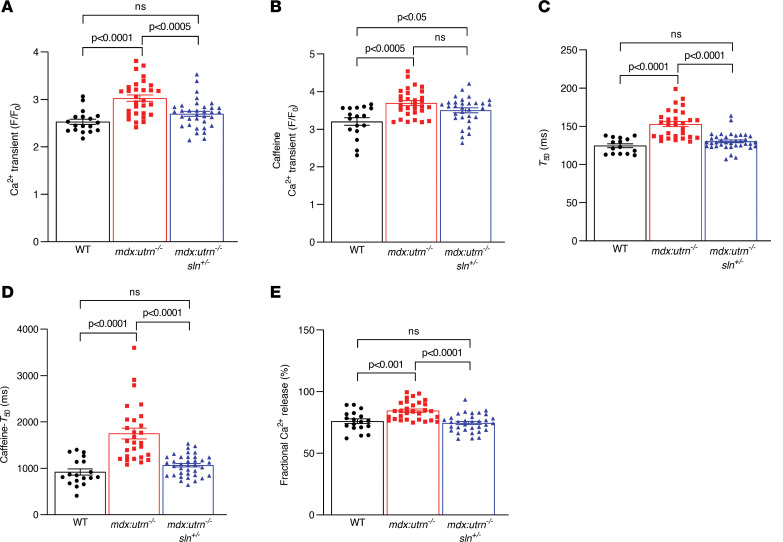
SR Ca^2+^ handling is improved in cardiomyocytes from *mdx:utrn^–/–^:sln^+/–^* mice. Summarized data for (**A**) twitch Ca^2+^ transients, (**B**) caffeine-induced Ca^2+^ transients, (**C**) the 50% decline in the duration (*T*_50_) of twitch Ca^2+^ transients, (**D**) *T*_50_ of caffeine-induced Ca^2+^ transients, and (**E**) fractional SR Ca^2+^ release obtained from cardiomyocytes isolated from wild-type (WT), *mdx:utrn^–/–^*, and *mdx:utrn^–/–^:sln^+/–^* mice. F/F_0_, ratio of the fluorescence over the basal diastolic fluorescence. The total number of myocytes shown was from 3 mice per genotype. Data were analyzed by ordinary 1-way ANOVA for multigroup comparisons. Values shown are means ± SE.

We next examined the Ca^2+^ handling properties of cardiomyocytes from WT, *mdx:utrn^–/–^*, and *mdx:utrn^–/–^:sln^+/–^* mice. The results showed significantly higher amplitudes of twitch Ca^2+^ transients (Figure 1A), increased SR Ca^2+^ content represented by increased caffeine-induced Ca^2+^ transient amplitudes (Figure 1B), decreased SR Ca^2+^ uptake represented by the increased time taken for the 50% decrease in twitch Ca^2+^ transients (*T*_50_; Figure 1C), decreased Ca^2+^ removal from the cytoplasm represented by increased *T*_50_ of caffeine-induced Ca^2+^ transients (Figure 1D), and defective RyR function represented by increased fractional Ca^2+^ release (Figure 1E) in myocytes from *mdx:utrn^–/–^* mice. On the other hand, in myocytes from *mdx:utrn^–/–^:sln^+/–^* mice, all of these parameters except for the caffeine-induced Ca^2+^ transients were restored to levels comparable to WT myocytes (Figure 1, A–E).

Western blot analyses showed that the SLN protein level, which was significantly higher in the ventricles of *mdx:utrn^–/–^* mice (Supplemental Figure 3, A and B), was reduced in the ventricles of *mdx:utrn^–/–^:sln^+/–^* mice and was similar to that of WT control ventricles. The protein levels of SERCA2a, phospholamban (PLN), RyR2, and calcineurin were unaltered and comparable in the ventricles of WT, *mdx:utrn^–/–^*, and *mdx:utrn^–/–^:sln^+/–^* mice (Supplemental Figure 3).

### Reducing SLN expression prevents Ca^2+^_m_ overload and improves mitochondrial function in the mdx:utrn^–/–^ myocardium.

**Figure 2:**
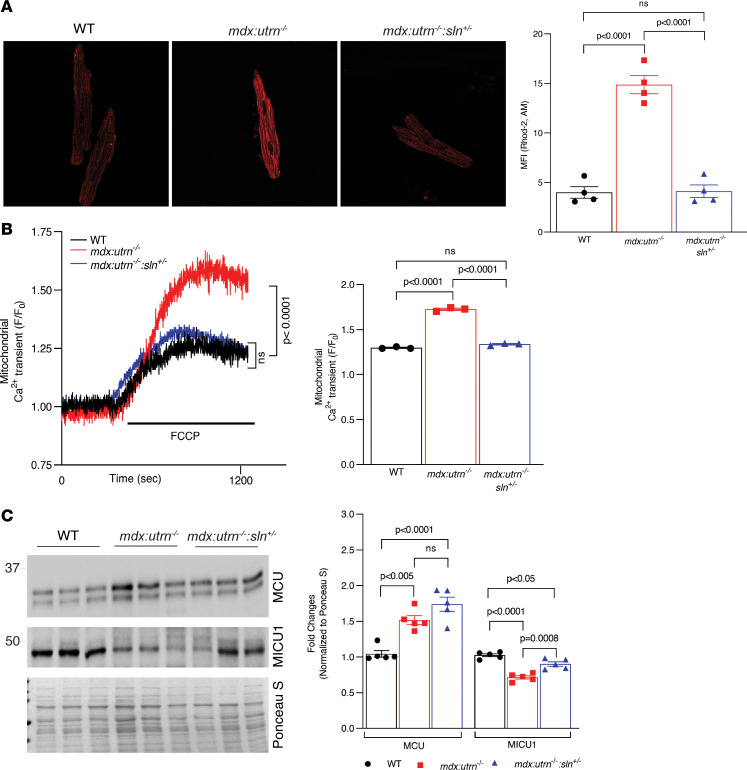
Ca^2+^_m_ handling is improved in the *mdx:utrn^–/–^:sln^+/–^* myocardium. (**A**) Representative confocal images of cardiomyocytes from WT, *mdx:utrn^–/–^*, and *mdx:utrn^–/–^:sln^+/–^* mice stained with reduced Rhod-2, AM (left panel), and summarized quantitation data showing the MFI (right panel). We used 10–25 myocytes per mouse heart. *n* = 4 mice per genotype. For all images, the original magnification is 60×. (**B**) Representative traces (left panel) and quantitation of F/F_0_ (right panel) showing Ca^2+^_m_ transients in cardiomyocytes from WT, *mdx:utrn^–/–^*, and *mdx:utrn^–/–^:sln^+/–^* mice. We used 5–12 myocytes per mouse heart. *n* = 3 mice per genotype. (**C**) Representative Western blots (left panel) and quantitation (right panel) showing MCU and MICU1 protein levels in the ventricles of WT, *mdx:utrn^–/–^*, and *mdx:utrn^–/–^:sln^+/–^* mice. For Western blotting, 10 μg of pro tein is loaded per well. *n* = 5 mice per genotype. Data were analyzed by ordinary 1-way ANOVA for multigroup comparisons. Values shown are means ± SE.

We next examined whether improving the SR Ca^2+^ handling via reducing SLN expression improves Ca^2+^_m_ cycling in dystrophic cardiomyocytes. Rhod-2, AM, a high-affinity Ca^2+^ indicator that selectively localizes to mitochondria, was used to determine the Ca^2+^_m_ content in cardiomyocytes using confocal imaging. The mean fluorescence intensity (MFI) of Rhod-2 was significantly higher in *mdx:utrn^–/–^* ventricular myocytes (Figure 2A), indicating increased Ca^2+^_m_ content. The MFI in *mdx:utrn^–/–^:sln^+/–^* ventricular myocytes, on the other hand, was significantly lower and comparable to that of WT controls (Figure 2A).

To further validate our findings, we examined Ca^2+^_m_ efflux, an indirect measurement of Ca^2+^_m_ content, as described before (12, 20). In brief, plasmalemmal Ca^2+^ influx was first blocked by treating myocytes with Li^+^ Tyrode’s solution devoid of Na^+^ and Ca^2+^, followed by tetracaine and thapsigargin treatment, which inhibit SR Ca^2+^ release and uptake, respectively. Myocytes were then treated with FCCP and oligomycin to release Ca^2+^ from mitochondria, resulting in an increase in cytosolic Ca^2+^ levels (referred to as Ca^2+^_m_ efflux or transients), which serves as an indirect measure of Ca^2+^_m_ content (Supplemental Figure 4). Ca^2+^_m_ efflux was significantly higher in myocytes from *mdx:utr^–/–^* mice, whereas it was significantly lower in myocytes from *mdx:utrn^–/–^:sln^+/–^* mice and comparable to WT controls (Figure 2B). These results indicate that Ca^2+^_m_ content was higher in myocytes from *mdx:utrn^–/–^* mice whereas reducing SLN expression prevented Ca^2+^_m_ overload in *mdx:utrn^–/–^* myocytes.

Western blot analyses showed that the protein levels of the leucine zipper and EF-hand containing transmembrane protein 1 (LETM1), mitochondrial Na^2+^/Ca^2+^ exchanger (NCLX), and canonical transient receptor potential 1 (TRPC1) were not different among the hearts of all 3 groups of mice (Supplemental Figure 5). On the other hand, mitochondrial Ca^2+^ uniporter (MCU) was significantly higher in the ventricles of both *mdx:utrn^–/–^* and *mdx:utrn^–/–^:sln^+/–^* mice compared with that of WT controls (Figure 2C). MICU1, an MCU subunit that regulates channel opening, was significantly lower in the *mdx:utrn^–/–^* ventricles than in the WT and *mdx:utrn^–/–^:sln^+/–^* ventricles (Figure 2C).

**Figure 3:**
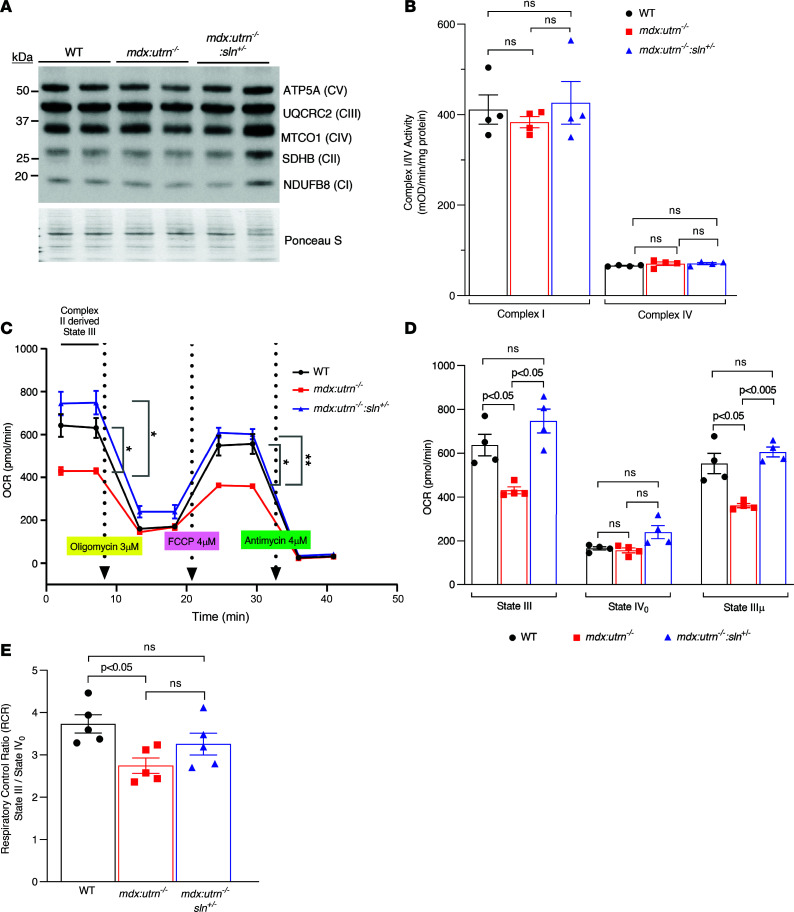
Mitochondrial respiration is improved in the *mdx:utrn^–/–^:sln^+/–^* myocardium. (**A**) Representative Western blots showing the protein levels of electron transport chain complexes I, II, III, IV, and V subunits in the ventricles of WT, *mdx:utrn^–/–^*, and *mdx:utrn^–/–^:sln^+/–^* mice. *n* = 4 mice per group. For Western blotting, 10 μg of protein is loaded per well. (**B**) Summary of quantification showing mitochondrial complex I and complex IV activities in the ventricular lysates. *n* = 4 mice per genotype. (**C**) Representative line graphs showing complex II–driven respiration measuring oxygen consumption rate (OCR) at basal levels and after treatment with oligomycin, FCCP, and antimycin in intact heart mitochondria purified from WT, *mdx:utrn^–/–^*, and *mdx:utrn^–/–^:sln^+/–^* mice. *P < 0.05; **P < 0.005. Quantification showing the (**D**) state III, state IV_0_, and state IIIμ respirations (*n* = 4 mice per genotype) and (**E**) RCR (*n* = 5 mice per genotype) in the intact heart mitochondria purified from WT, *mdx:utrn^–/–^*, and *mdx:utrn^–/–^:sln^+/–^* mice. Sample triplicates were used for Seahorse assays. Data were analyzed by ordinary 1-way ANOVA for multigroup comparisons. Values shown are means ± SE.

Ca^2+^_m_ levels are known to regulate mitochondrial respiration. Therefore, we next examined the expression levels and activities of various electron complex chain subunits. Western blot analyses showed that the protein levels of complexes I (NDUF88), II (SDHB), III (UQCRC2), IV (MTCO1), or V (ATP5A) subunits in the ventricles of WT, *mdx:utrn^–/–^*, and *mdx:utrn^–/–^:sln^+/–^* mice were not significantly different (Figure 3A). Complex I and complex IV activity in ventricular lysates from WT, *mdx:utrn^–/–^*, and *mdx:utrn^–/–^:sln^+/–^* mice were comparable and not significantly different (Figure 3B).

Complex II dysfunction has been reported in many neurodegenerative diseases and aging (21). We, therefore, examined complex II respiration in intact mitochondria prepared from fresh ventricular tissues using the Seahorse XFe24 flux analyzer. Complex II–driven respiration was significantly lower in *mdx:utrn^–/–^* ventricular mitochondria compared with that of WT controls. On the other hand, complex II–driven respiration was significantly improved in *mdx:utrn^–/–^:sln^+/–^* ventricular mitochondria and comparable to WT controls (Figure 3C). The maximal rates of state III (ADP stimulated) and state IIIμ (FCCP stimulated) respiration were significantly reduced in *mdx:utrn^–/–^* ventricular mitochondria. However, the lower state IIIμ respiration indicates that the FCCP concentration may have had an inhibitory effect on the respiratory function in these experiments. Despite this effect, state III and IIIμ respirations were significantly higher and comparable to WT controls in *mdx:utrn^–/–^:sln^+/–^* ventricular mitochondria (Figure 3D). The respiratory control ratio (RCR), which represents mitochondrial health, was significantly lower in *mdx:utrn^–/–^* ventricles compared with WT controls. Although not statistically significant, RCR was higher in the mitochondria of *mdx:utrn^–/–^:sln^+/–^* ventricles (Figure 3E).

### Reducing SLN expression prevents mitochondrial membrane potential loss in dystrophic ventricular myocytes.

**Figure 4:**
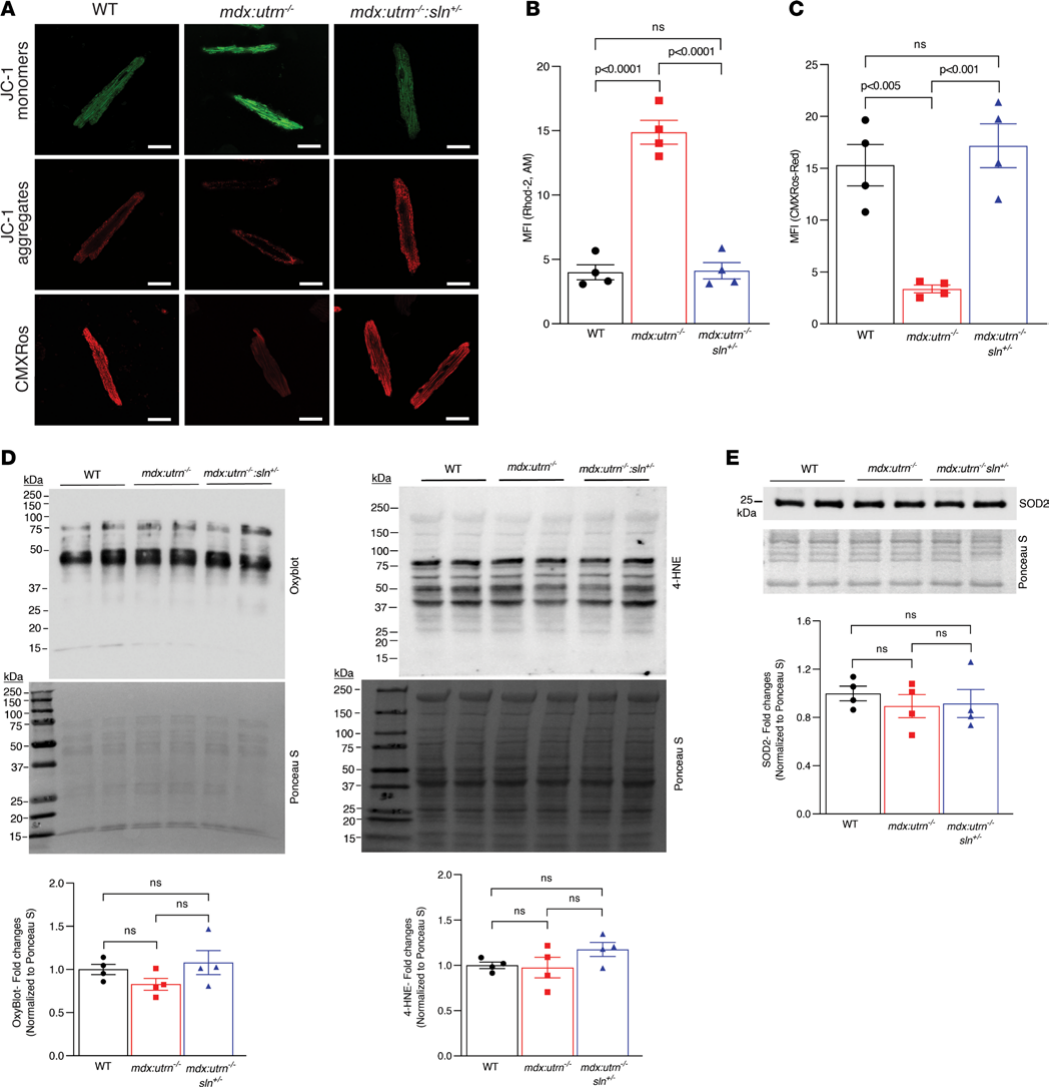
The ΔΨm is improved in the ventricular myocytes of *mdx:utrn^–/–^:sln^+/–^* mice. (**A**) Representative confocal images of ventricular myocytes stained with either JC-1 (15 μM) or CMXRos (100 nM). Scale bar = 25 μm. Original magnification is 60×. Quantification showing the (**B**) MFI ratio of red and green of JC-1 and (**C**) MFI of CMXRos red as fold-changes in WT, *mdx:utrn^–/–^*, and *mdx:utrn^–/–^:sln^+/–^* ventricular myocytes. We used 10–25 myocytes per mouse heart. *n* = 4 mice per genotype. (**D**) Representative OxyBlot and 4-HNE Western blots (top) and quantitation (bottom) showing the levels of carbonylated proteins and lipid peroxidation in the total protein extracts prepared from the ventricles of WT, *mdx:utrn^–/–^*, and *mdx:utrn^–/–^:sln^+/–^* mice. *n* = 4 mice per genotype. (**E**) Representative Western blot (top) and quantitation (bottom) showing the levels of SOD2 in the ventricles of WT, *mdx:utrn^–/–^*, and *mdx:utrn^–/–^:sln^+/–^* mice. *n* = 4 mice per genotype. For Western blotting, 10 μg of protein is loaded per well. Data were analyzed by ordinary 1-way ANOVA for multigroup comparisons. Values shown are means ± SE.

Mitochondrial dysfunction is normally associated with either loss of membrane potential or elevated oxidative stress. We first assessed mitochondrial membrane potential (ΔΨm) in isolated cardiomyocytes using the ratiometric dye JC-1, as well as MitoTracker Red CMXRos that accumulates in mitochondria in live cells depending upon membrane potential (22). The mean red/green fluorescence intensity ratio of JC-1 in myocytes from *mdx:utrn^–/–^* mice was significantly lower, indicating mitochondrial depolarization, whereas it was significantly higher in the *mdx:utrn^–/–^:sln^+/–^* myocytes (Figure 4, A and B). Similarly, the MFI of MitoTracker Red CMXRos in myocytes from *mdx:utrn^–/–^* mice was significantly lower whereas the MFI was higher in *mdx:utrn^–/–^:sln^+/–^* myocytes and comparable to that of WT myocytes (Figure 4, A and C). These data indicate that there is a loss of ΔΨm in myocytes from *mdx:utrn^–/–^* mice and that reducing SLN expression prevents ΔΨm loss.

We next examined whether oxidative stress contributes to the ΔΨm loss in myocytes from *mdx:utrn^–/–^* mice by measuring protein carbonylation and lipid peroxidation using OxyBlot and 4-hydroxynonenal (4-HNE) staining, respectively. The protein carbonylation and lipid peroxidation in total protein extract (Figure 4D) as well as in the purified mitochondrial fractions (Supplemental Figure 6) were similar among WT, *mdx:utrn^–/–^*, and *mdx:utrn^–/–^:sln^+/–^* ventricles. The protein level of manganese-dependent superoxide dismutase 2 (MnSOD/SOD2), a major mitochondrial antioxidant enzyme, was also similar among WT, *mdx:utrn^–/–^*, and *mdx:utrn^–/–^:sln^+/–^* ventricles (Figure 4E).

### Reducing SLN expression restores mitochondrial morphology in dystrophic myocardium.

**Figure 5:**
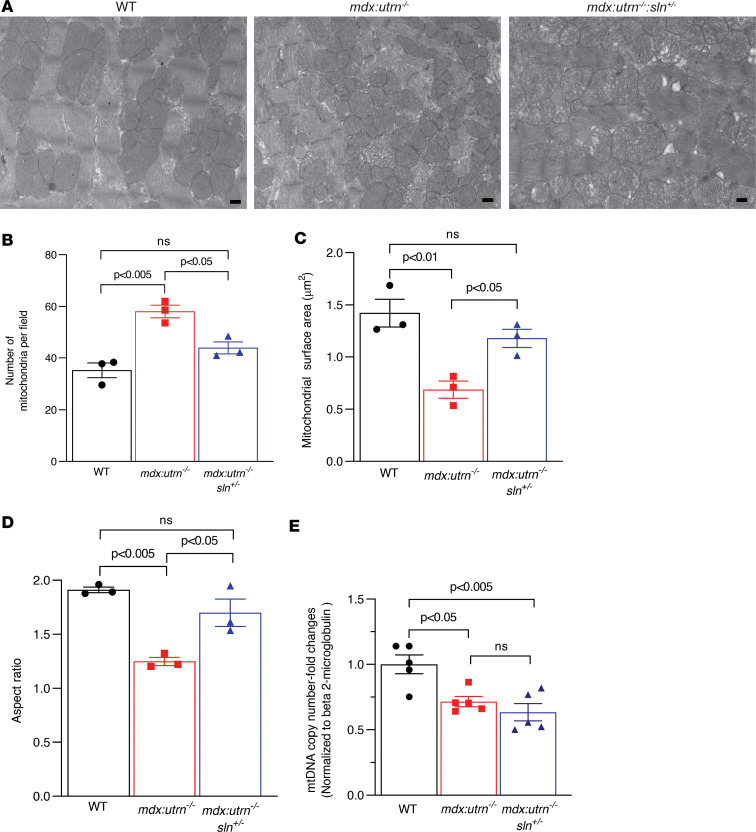
Improved mitochondrial structure in *mdx:utrn^–/–^:sln^+/–^* ventricles. (**A**) Representative transmission electron micrographs of ventricular sections from WT, *mdx:utrn^–/–^*, and *mdx:utrn^–/–^:sln^+/–^* mice. Scale bar = 500 nm. Original magnification is 6,300×. Quantification showing (**B**) mitochondrial number, (**C**) mitochondrial surface area (μm^2^), and (**D**) aspect ratio of mitochondria. We randomly selected 6 to 8 fields at 6,300× original magnification and analyzed 130 to 150 mitochondria/mouse heart. *n* = 3 mice per genotype. (**E**) qPCR analysis showing mitochondrial copy number. *n* = 5 mice per genotype. Data were analyzed by ordinary 1-way ANOVA for multigroup comparisons. Values shown are means ± SE.

We then measured mitochondrial number, surface area, and aspect ratio in ventricular sections using transmission electron microscopy (TEM) to determine whether reducing Ca^2+^_m_ load affected mitochondrial morphology in *mdx:utrn^–/–^:sln^+/–^* myocardium (Figure 5A). The number of mitochondria in *mdx:utrn^–/–^* ventricles was significantly higher than in WT and *mdx:utrn^–/–^:sln^+/–^* ventricles (Figure 5B). Furthermore, mitochondria from *mdx:utrn^–/–^* ventricles had a smaller surface area (Figure 5C) and aspect ratio (Figure 5D). The mitochondrial surface area and aspect ratio in the *mdx:utrn^–/–^:sln^+/–^* ventricles, on the other hand, were significantly higher and comparable to WT ventricles (Figure 5, C and D). The mitochondrial copy number assessed by quantitative PCR (qPCR), however, was significantly low in the ventricles of both *mdx:utrn^–/–^* and *mdx:utrn^–/–^:sln^+/–^* mice (Figure 5E).

### Reducing SLN expression improves both SR and non-SR-associated mitochondrial structure in dystrophic myocardium.

**Figure 6:**
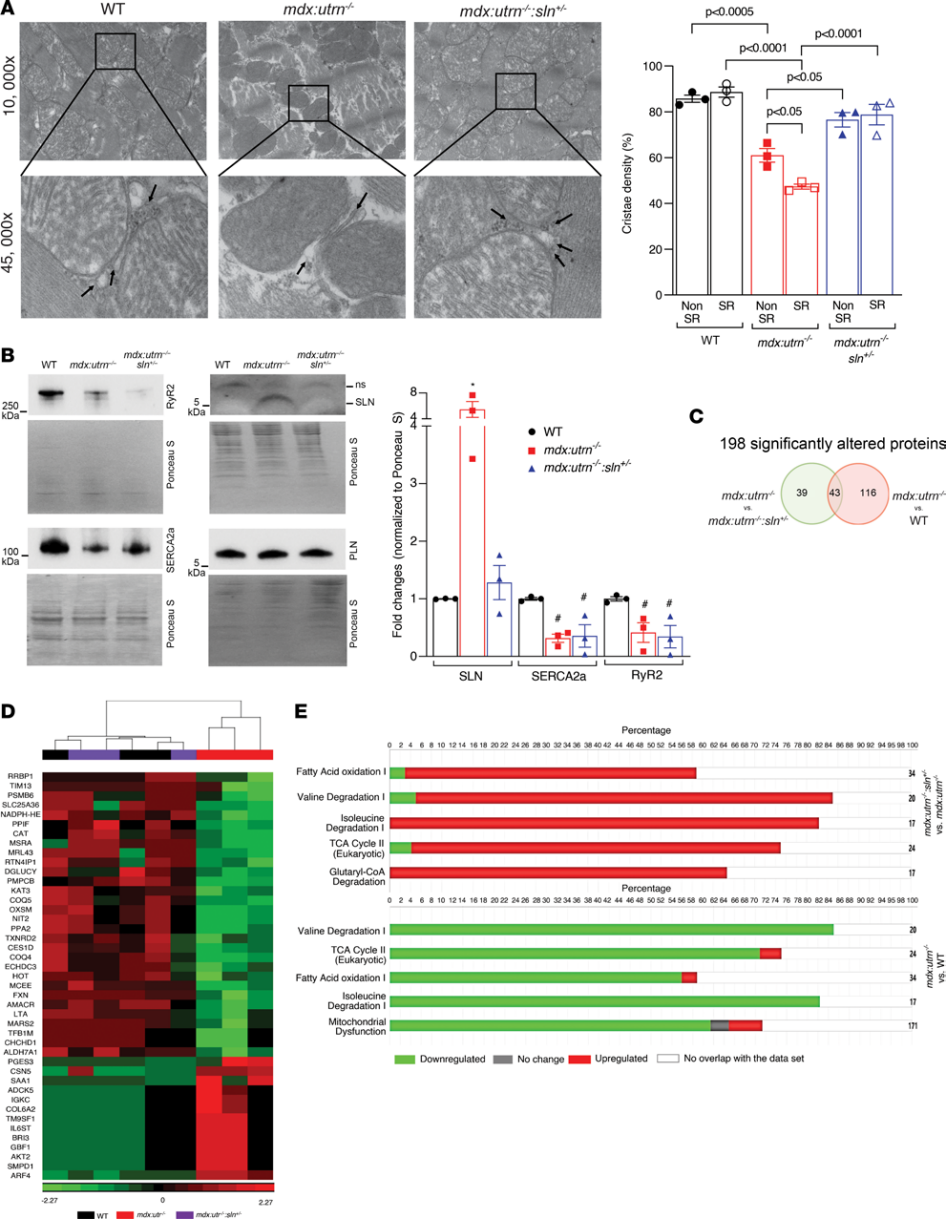
Improved mitochondrial structure and MAMs in *mdx:utrn^–/–^:sln^+/–^* ventricles. (**A**) Representative transmission electron micrographs of ventricular sections from WT, *mdx:utrn^–/–^*, and *mdx:utrn^–/–^:sln^+/–^* mice (left panel). The 45,000× original magnification shows the SR-mitochondrial junctions (indicated by arrows) and loss of cristae in the SR-associated mitochondria. Quantification (right panel) showing cristae density in SR-associated and non-SR-associated mitochondria. We randomly selected 6 to 8 fields at 10,000× original magnification and analyzed approximately 150 mitochondria/genotype (40–60 mitochondria/mouse heart) for cristae density measurements. *n* = 3 mice per genotype. Data were analyzed by ordinary 1-way ANOVA for multigroup comparisons. Values shown are means ± SE. (**B**) Representative Western blots (left panel) and quantitation (right panel) showing RyR2, SERCA2a, SLN, and PLN protein levels in the MAMs purified from the myocardium of WT, *mdx:utrn^–/–^*, and *mdx:utrn^–/–^:sln^+/–^* mice. We pooled 4–5 ventricles for each MAM preparation. *n* = 3 MAM preparation/genotype. For Western blotting, 10–20 μg of MAM protein is loaded per well. Data were analyzed by ordinary 1-way ANOVA for multigroup comparisons. Values shown are means ± SE. *P < 0.01 vs. WT and *mdx:utrn^–/–^:sln^+/–^*; ^#^*P* < 0.05 vs. WT. (**C**) Venn diagram showing significantly altered MAM proteins between *mdx:utrn^–/–^* and WT and *mdx:utrn^–/–^:sln^+/–^* and *mdx:utrn^–/–^* mice. (**D**) Heatmap of 43 MAM proteins that are well clustered between WT and *mdx:utrn^–/–^:sln^+/–^* but differentially expressed in *mdx:utrn^–/–^* mice. (**E**) Horizontal stacked bar chart comparing the top canonical pathways either up- or downregulated in the MAMs between *mdx:utrn^–/–^:sln^+/–^* and *mdx:utrn^–/–^* and between *mdx:utrn^–/–^* and WT mice with a *P* value cutoff of 0.05.

We next examined the mitochondrial structure, particularly cristae density, in the ventricles of WT, *mdx:utrn^–/–^*, and *mdx:utrn^–/–^:sln^+/–^* mice using TEM (Figure 6A). Quantitation showed that there was a significant reduction in cristae density in the mitochondria from *mdx:utrn^–/–^* ventricles. These reductions in cristae density were more pronounced in SR-associated mitochondria than in non-SR-associated mitochondria. The cristae density was significantly higher in both SR-associated and non-SR-associated mitochondria from *mdx:utrn^–/–^:sln^+/–^* ventricles than in *mdx:utrn^–/–^* ventricles (Figure 6A).

We next examined the SR-mitochondrial junctions by analyzing the mitochondria-associated membranes (MAMs). The purity of the MAMs was verified by Western blot analysis by probing for SR, mitochondria, nuclei, and cytoplasm-specific proteins (Supplemental Figure 7). Western blot analyses of SR-mitochondria interface–associated proteins showed that SLN levels were high whereas the levels of SERCA2a and RyR2 were significantly low in the MAMs of *mdx:utrn^–/–^* ventricles (Figure 6B). In the MAMs of *mdx:utrn^–/–^:sln^+/–^* ventricles, the SLN level was significantly reduced, whereas SERCA2a and RyR2 levels remained low and not significantly different from those of *mdx:utrn^–/–^* ventricles (Figure 6B). PLN levels were unaltered among the MAMs of WT, *mdx:utrn^–/–^*, and *mdx:utrn^–/–^:sln^+/–^* ventricles (Figure 6B).

Proteomic profiling with liquid chromatography-tandem mass spectrometry (LC-MS/MS) of MAMs identified a total of 2,300 proteins across WT, *mdx:utrn^–/–^*, and *mdx:utrn^–/–^:sln^+/–^* mouse groups. Gene Ontology (GO) term mapping analysis was performed on the batch-corrected total proteins identified to parse the list based on their subcellular localization. The total number of proteins, as well as proteins pertaining to mitochondrial and SR localizations, were not significantly different among the 3 groups (Supplemental Figure 8). However, there were 159 and 82 individual proteins that differed significantly (*P* < 0.05) between WT and *mdx:utrn^–/–^* mice and between *mdx:utrn^–/–^* and *mdx:utrn^–/–^:sln^+/–^* mice, respectively. As shown by the Venn diagram (Figure 6C), there were 43 proteins altered in both comparisons, i.e., WT versus *mdx:utrn^–/–^* MAMs as well as *mdx:utrn^–/–^* versus *mdx:utrn^–/–^:sln^+/–^* MAMs (Figure 6D). These included 30 proteins that were downregulated and 13 proteins that were upregulated in the MAMs of *mdx:utrn^–/–^* ventricles compared with those of WT mice. All 43 proteins were reversibly altered in *mdx:utrn^–/–^* versus *mdx:utrn^–/–^:sln^+/–^* MAMs. Hierarchical clustering of these 43 shared proteins revealed restoration to WT levels in the MAMs of *mdx:utrn^–/–^:sln^+/–^* ventricles, such that *mdx:utrn^–/–^:sln^+/–^* and WT MAMs represented a single cluster, indistinguishable from one another and clustered apart from *mdx:utrn^–/–^* MAMs (Figure 6D). Ingenuity Pathway Analysis (IPA; QIAGEN) of the significantly altered proteins (*P* = 0.05) revealed that metabolic pathways involved in mitochondrial function, such as fatty acid oxidation I and TCA cycle II, were downregulated in the MAMs of *mdx:utrn^–/–^* ventricles whereas these same pathways were upregulated in the MAMs of *mdx:utrn^–/–^:sln^+/–^* ventricles (Figure 6E).

## Discussion

Abnormal Ca^2+^_i_ cycling is one of the main disease-causing mechanisms in DMD (4–8). Chronic accumulation of cytoplasmic Ca^2+^ could force mitochondria to uptake more Ca^2+^ and increase Ca^2+^_m_ concentration, which further contributes to mitochondrial dysfunction and myofiber necrosis (7, 10, 11). In support of this notion, preventing mitochondrial dysfunction is shown to partially rescue the muscular dystrophy phenotype (23–25). However, the molecular mechanism(s) that contribute to Ca^2+^_m_ overload and mitochondrial dysfunction in dystrophic myocardium is not fully understood. The findings from the current study indicate that SLN upregulation contributes substantially to mitochondrial dysfunction in the myocardium of *mdx:utrn^–/–^* mice. Our studies specifically demonstrated that reducing SLN expression can (i) improve SR Ca^2+^ handling and prevent Ca^2+^_m_ overload, (ii) prevent ΔΨm loss, (iii) improve mitochondrial respiration, (iv) reduce mitochondrial cristae loss, and (v) improve SR-mitochondria interaction in the myocardium of *mdx:utrn^–/–^* mice.

Consistent with the *mdx* model (18), cardiomyocytes from *mdx:utrn^–/–^* mice show dysregulated Ca^2+^_i_ handling, as evidenced by increased twitch Ca^2+^ transients, decreased SR Ca^2+^ uptake, increased SR Ca^2+^ leak, defective Ca^2+^ removal from the cytosol, and SR Ca^2+^ overload. In addition, we found increased levels of Ca^2+^_m_ content in dystrophic cardiomyocytes. Intriguingly, reducing SLN expression not only improved the SR Ca^2+^ cycling but also reduced Ca^2+^_m_ overload in dystrophic myocytes. These findings suggest that improving SERCA function via SLN reduction can prevent chronic accumulation of cytoplasmic Ca^2+^ and thereby reduce Ca^2+^_m_ uptake in the dystrophic myocardium. In support of this, both reduction and ablation of SLN improved SERCA function, as well as reduced Ca^2+^_m_ load in skeletal muscles of *mdx* mice (19).

Molecular mechanisms causing Ca^2+^_m_ overload in dystrophic cardiomyocytes are not fully understood. NCLX, which is involved in Ca^2+^_m_ efflux (26), is significantly higher in the *mdx* heart mitochondria (27). However, NCLX protein levels in the hearts of *mdx:utrn^–/–^* mice remain unchanged. In addition, the protein levels of LETM1, a mitochondrial Ca^2+^/H^+^ antiporter involved in Ca^2+^_m_ sequestration at a low increase in Ca^2+^ (28), were not altered in the myocardium of *mdx:utrn^–/–^* mice compared with those of WT controls. Furthermore, reducing SLN expression did not affect the expression levels of these proteins in the dystrophic myocardium, suggesting that these proteins may not contribute to the Ca^2+^_m_ overload in the cardiomyocytes of *mdx:utrn^–/–^* mice.

MCU, a Ca^2+^ selective channel (29), and its modulator, MICU1 (30, 31), are shown to be expressed at higher levels in *mdx* hearts and have been linked to increased Ca^2+^_m_ uptake (32, 33). In *mdx:utrn^–/–^* myocardium, we found elevated levels of MCU but significantly lower levels of MICU1 (Figure 2C). Despite the low Ca^2+^_m_ content, MCU levels remained high in the *mdx:utrn^–/–^*:*sln^+/–^* ventricles whereas MICU1 levels increased. MICU1 has been shown to play a dual role in MCU regulation, acting as an inhibitor at low cytoplasmic Ca^2+^ concentration and an activator at high cytoplasmic Ca^2+^ concentration (31, 34). We, therefore, speculate that increasing MCU levels could result in increased Ca^2+^_m_ content in dystrophic myocytes. On the other hand, changes in MICU1 levels could be compensatory in *mdx:utrn^–/–^* and *mdx:utrn^–/–^*:*sln^+/–^* myocytes as a means of normalizing Ca^2+^_m_ load. The functional relevance of the differential expression of MCU and MICU1 in regulating Ca^2+^_m_ uptake in *mdx:utrn^–/–^* and *mdx:utrn^–/–^*:*sln^+/–^* myocardium, however, remains to be expanded by future studies.

Several studies have reported impaired oxidative phosphorylation, elevated reactive oxygen species (ROS) generation, impaired mitochondrial respiration, and reduced ATP production in skeletal and cardiac muscles of *mdx* mice and skeletal muscle biopsies from patients with DMD (12, 13, 33, 35–38). We found no difference in the protein levels of electron transport chain protein subunits or individual mitochondrial complex activities in the hearts of *mdx:utrn^–/–^* mice. Our findings are also consistent with previously reported unchanged mitochondrial complex activities in the skeletal muscles of *mdx:utrn^–/–^* mice (39). Furthermore, there was no difference in protein oxidation and carbonylation, and the protein level of MnSOD suggests that oxidative stress may not play a significant role in mitochondrial dysfunction in *mdx:utrn^–/–^* hearts. However, to validate this notion, direct measurement of ROS, measurement of other antioxidants, and NADPH oxidase pathways need to be studied in these hearts. In the absence of oxidative stress and unchanged complex activities, loss of ΔΨm might significantly contribute to decreased mitochondrial respiration in the cardiomyocytes of *mdx:utr^–/–^* mice. In support of this, there is a decrease in ΔΨm and impaired mitochondrial respiration in the *mdx:utrn^–/–^* cardiomyocytes. Excessive accumulation of Ca^2+^ in mitochondria leads to functional impairments, which could lead to decreased ΔΨm (40). Thus, SLN reduction improved ΔΨm and mitochondrial function in *mdx:utrn^–/–^* cardiomyocytes by reducing Ca^2+^_m_ overload.

In skeletal muscles, SLN overexpression is shown to uncouple the SERCA pump as well as induce mitochondrial biogenesis and enhance energy metabolism (41). However, in dystrophic myocardium, SLN overexpression could uncouple the SERCA pump from Ca^2+^ transport, resulting in the accumulation of Ca^2+^ in the cytoplasm and increased ATP utilization. This could further increase the cytoplasmic Ca^2+^ load and cause an increase in energy demand, leading to increased Ca^2+^_m_ uptake, loss of ΔΨm, and mitochondrial dysfunction. This is supported by the increased loss of mitochondrial cristae, a membrane hub where most of the respiratory complexes embed to account for oxidative phosphorylation and ATP synthesis in the *mdx:utrn^–/–^* myocytes. In support of this idea, reducing SLN expression improved SERCA function, reduced Ca^2+^_m_ overload, prevented the loss of ΔΨm, and improved mitochondrial cristae structure and respiration in dystrophic myocardium. Reducing or abolishing SLN expression also prevents Ca^2+^_m_ overload and improves metabolism in the muscles of *mdx* mice (19). Thus, reducing SLN expression in dystrophic muscles is rather beneficial and improves mitochondrial function.

Electron micrographs of ventricular tissues from *mdx:utrn^–/–^* mice revealed a large number of smaller mitochondria, as evidenced by an increased number of mitochondria with a smaller surface area, indicating impaired mitochondrial dynamics. Furthermore, mitochondrial copy number is significantly reduced in *mdx:utrn^–/–^* ventricles, indicating defects in mitochondrial fission and fusion cycles (Figure 5E). Although SLN reduction did not normalize mitochondrial copy number, it did improve mitochondrial number, surface area, and aspect ratio in *mdx:utrn^–/–^* ventricles. These findings suggest that reducing SLN expression could partially restore mitochondrial dynamics. It is also worth mentioning that SLN reduction restores autophagy and mitochondrial dynamics in dystrophic dog myotubes (42). Our future studies will address the molecular mechanisms associated with mitochondrial dynamics in the *mdx:utrn^–/–^:sln^+/–^* myocardium.

A growing number of studies suggest that a proper ER/SR-mitochondria interface is required for mitochondrial function (43). Recent studies also indicate that ER/mitochondrial junctions are disrupted in the heart (33) and muscles (44) of *mdx* mice. However, there are no studies on the interaction of SR and mitochondria, though SR is the major regulator of cytosolic Ca^2+^ in the heart during excitation-contraction coupling. Our findings show that cristae density loss in SR-associated mitochondria is significantly greater than in non-SR-associated mitochondria in *mdx:utrn^–/–^* ventricles (Figure 6A). Furthermore, we found increased levels of SLN, decreased levels of SR Ca^2+^ uptake (SERCA2a) and release (RyR) proteins, as well as decreased levels of proteins involved in mitochondrial metabolism in the MAMs of *mdx:utrn^–/–^* myocardium. These findings suggest that SR-mitochondria interactions are significantly impaired in the *mdx:utrn^–/–^* myocardium. The increased SERCA2a/SLN ratio in the MAMs of *mdx:utrn^–/–^* myocardium suggests that SLN could directly inhibit the SERCA pump in MAMs. In addition, SLN may enhance the inhibitory effect of PLN (45), another key regulator of SERCA2a that is unaltered in the MAMs of *mdx:utrn^–/–^* myocardium. Thus, SLN enrichment in the MAMs, which has not been previously reported to our knowledge, could result in decreased SERCA function and increased Ca^2+^ accumulation at the SR-mitochondria junction, forcing the mitochondria to uptake more Ca^2+^ and causing mitochondrial dysfunction and damage. The fact that SLN haploinsufficiency results in reduced levels of SLN in the MAMs, reduced Ca^2+^_m_ content, and improved mitochondrial structure and function in the ventricles of *mdx:utrn^–/–^:sln^+/–^* mice validates this idea. It is also worth noting that the total SERCA2a, PLN, and RyR levels are unaffected in the *mdx:utrn^–/–^* ventricles, and SLN reduction or ablation does not affect the expression of these proteins (Supplemental Figure 3) (17).

Proteomic analyses show the enrichment of proteins involved in the metabolic pathways and mitochondrial function in the MAMs of *mdx:utrn^–/–^:sln^+/–^* myocardium. Although elucidation of the underlying changes in MAMs that drive the salutary effects of SLN reduction in the setting of DMD is beyond the scope of this study, results gleaned from proteomic profiling of MAMs provide valuable insights for future mechanistic investigation. Frataxin (FXN), for instance, which functions in regulating mitochondrial iron transport and respiration, was reduced in dystrophic MAMs and restored by SLN reduction (Figure 6D). Lack of FXN is known to cause mitochondrial dysfunction and Friedreich’s ataxia, a recessive neurodegenerative disorder commonly associated with hypertrophic cardiomyopathy (46). Thus, there may be a potential association between FXN reduction in the MAMs and mitochondrial dysfunction and cardiomyopathy in DMD. Similarly, mitochondrial methionyl-tRNA synthetase 2 protein, which stimulates Ca^2+^_m_ influx via direct interaction with MCU (47), was restored to normal levels in *mdx:utrn^–/–^:sln^+/–^* MAMs (Figure 6D) and might thus contribute to the observed improvements in Ca^2+^_m_ handling in the ventricles of *mdx:utrn^–/–^:sln^+/–^* mice. Likewise, several proteins that were diminished in dystrophic MAMs and rescued by SLN knockdown could contribute to observed improvements in mitochondrial structure and function. Our future studies are aimed at dissecting the roles of these altered proteins in DMD, particularly the normative effects of SLN depletion and how SLN orchestrates mitochondrial dynamics.

In summary, our data reveal that reducing SLN expression is sufficient to restore cardiac SERCA function and subsequently Ca^2+^_i_ handling in dystrophic myocardium. These changes can further improve SR/mitochondrial interaction, prevent Ca^2+^_m_ overload and loss of ΔΨm, and reduce mitochondrial structural damage and adverse cardiac remodeling, thereby delaying the progression of cardiomyopathy. Current findings not only advance our understanding of the molecular mechanisms associated with mitochondrial dysfunction in dystrophic myocardium but also add a new dimension to the therapeutic exploration of reducing or inhibiting SLN function in preventing cardiomyopathy in DMD.

## Methods

### Sex as a biological variable.

We used only 3- to 4-month-old male mice for this study because of the X-linked inheritance of DMD and its potential relevance to human patients, as well as the difficulty in breeding these mice to obtain a large number of mice of both sexes.

### Mouse models.

The *mdx:utrn^–/–^:sln^+/–^* mice were generated by crossing *mdx:utrn^+/−^* mice to *sln*^–/–^ mice as described before (17). These mice were crossed for 5 generations to obtain the *mdx:utrn^+/–^:sln^+/–^* mice in an isogenic background. The male and female *mdx:utrn^+/–^:sln^+/–^* mice were then crossed to generate the *mdx:utrn*^–/–^ and *mdx:utrn*^–/–^*:sln^+/–^* mice. The genotypes of the mice were identified by PCR using previously published sequences (48, 49). Mice were kept under a 12-hour light/12-hour dark cycle with a temperature of 22°C–24°C and 60%–70% humidity and fed ad libitum under a normal chow diet. Animal numbers were predetermined based on pilot studies, and sample sizes were similar to those generally employed in the field. No samples, mice, or data points were excluded from the data analysis. Animals were not randomized except for the genotypes. For echocardiography, genotypes were masked.

### Histology.

Five-micron, paraffin-embedded myocardial sections were used for standard PSR staining. The collagen fibers stained in red by PSR were calculated using the NIH ImageJ 1.43u program.

### Echocardiography.

Echocardiography was performed on anesthetized (2% isoflurane) mice using a high-resolution ultrasound machine VisualSonic/Vevo 3100 system with a high-frequency transducer (MXT400) (50). The LV systolic function was assessed by measuring LV interventricular septal thicknesses, LV internal dimensions, and posterior wall thicknesses at diastole and systole (IVSd, LVIDd, PWd, and IVSs, LVIDs, PWs, respectively) from M-mode images at the level of the papillary muscles. LV EF and LV FS were calculated using the Vevo 3100 software. Additionally, LV diastolic function was evaluated using pulse wave Doppler by imaging transmitral inflow Doppler via an apical 4-chamber view. The Doppler indexes include the E/A ratio, deceleration time of early filling of mitral inflow, isovolumetric relaxation time (IVRT), and isovolumetric contraction time (IVCT). Doppler parameters such as the E/A ratio and MPI (MPI = [IVRT + IVCT]/ejection time) were calculated to determine the diastolic function and overall myocardial performance.

### Ca^2+^ transient measurements.

Cardiomyocytes were enzymatically isolated from mouse ventricles following Langendorff heart perfusions as described previously (51, 52). Ca^2+^ transient amplitude and SR Ca^2+^ content were measured in isolated cardiomyocytes using Ca^2+^ indicator Fluo-4, AM (Molecular Probes F14201), at 34°C–36°C, as described earlier (18). Briefly, myocytes were loaded with Fluo-4, AM, and field-stimulated at 0.5 Hz to maintain consistent SR Ca^2+^ load. The fluorescence was excited at ~485 nm, and the emissions were measured at ~530 nm, using a Nikon Eclipse TE200 inverted microscope. Fluorescence intensity was measured as the ratio of F/F_0_. The amplitude of the caffeine-induced (10 mmol/L) Ca^2+^ transient was used as a measure of total SR Ca^2+^ content. Fractional SR Ca^2+^ release was calculated by dividing the height of the last twitch transient by the height of the caffeine transient.

### Ca^2+^_m_ efflux.

The Ca^2+^_m_ efflux was determined as described previously (12, 20). Briefly, cardiomyocytes were loaded with Fluo-4, AM; perfused with normal Tyrode’s; and stimulated at 0.5 Hz to delineate healthy cells, followed by perfusion with Li^+^ Tyrode’s (free of Ca^2+^ and Na^+^ and supplemented with glucose) for 30 seconds to prevent Ca^2+^ release/influx via NCLX and Ca^2+^ entry across the plasma membrane. Later, myocytes were perfused with Li^+^ Tyrode’s containing 1 mM tetracaine and 1 μM thapsigargin for 30 seconds to block both SR Ca^2+^ uptake and release. At this point, myocytes were perfused with 2 μM FCCP and 1 μg/mL oligomycin for 30 seconds along with tetracaine and thapsigargin to deplete the Ca^2+^_m_. This Ca^2+^_m_ release is observed as a slight elevation of cytosolic Ca^2+^ and measured as Ca^2+^_m_ efflux/transients (Supplemental Figure 4). The F/F_0_ was evaluated as relative Ca^2+^_m_ content.

### Confocal imaging and Ca^2+^_m_ measurements.

The Ca^2+^ content of mitochondria in isolated myocytes was evaluated using Rhod-2, AM (R1245MP, Invitrogen). According to the manufacturer’s protocols, Rhod-2, AM, was reduced just before the experiment using sodium borohydride (NaBH_4_) to further enhance mitochondrial localization. Myocytes were loaded with 5 μM reduced Rhod-2, AM, and incubated at 37°C or 30 minutes, followed by three 10-minute washes. Images were captured using the Nikon A1R confocal microscope at the Rutgers-Confocal Imaging Core Facility, New Jersey Medical School (NJMS), and the red fluorescence intensity was calculated using NIH ImageJ software.

### Measurements of ΔΨm.

Myocytes were loaded with either 15 μM JC-1 (T3168, Invitrogen) or 100 nM CMXRos (M7512, Invitrogen) and incubated at 37°C for 30 minutes followed by three 10-minute washes to remove the excess dye, according to manufacturer’s protocol. Later the images were captured using the Nikon A1R confocal microscope at the Rutgers-Confocal Imaging Core Facility, NJMS. The images were then analyzed using NIH ImageJ software to quantify the red/green ratio of the MFI for JC-1 and red MFI for CMXRos.

### Purification of MAMs.

MAMs were purified from fresh ventricular tissues following previously established procedures (53, 54). We pooled 4–5 ventricles for each preparation. In brief, ventricles were washed with ice-cold phosphate-buffered saline (PBS), homogenized in an isolation buffer (225 mM mannitol, 75 mM sucrose, 0.1% bovine serum albumin, 10 mM HEPES/Tris, 100 μM EGTA/Tris, pH 7.4) containing protease inhibitors, and centrifuged at 600*g* for 10 minutes to pellet down the unbroken cells and nucleus. The supernatant was centrifuged 2 more times, and the final supernatant collected was centrifuged at 10,000*g* for 10 minutes to pellet down the crude mitochondria. The supernatant containing microsomal (SR/ER) and cytosolic proteins was collected separately and stored at 4°C until further processing. The crude mitochondrial pellet was washed twice to remove any microsomal contamination. Further, the crude mitochondria were suspended in a small volume of isolation buffer, overlaid on Percoll medium (Percoll buffer containing mannitol [400 mg] and sucrose [250 mg] in isolation buffer), and centrifuged at 100,000*g* for 1 hour. The resulting centrifugation forms a top layer of MAM fraction and a bottom layer of pure mitochondria above the Percoll pellet. The MAM and mitochondrial fractions were collected into separate tubes and restored to 10 times the volume with an isolation buffer. Both fractions collected were then subjected to 6,300*g* for 10 minutes to prevent contamination of MAMs in the mitochondrial fraction and mitochondrial contamination in the MAMs. The final mitochondrial pellet was resuspended in an isolation buffer. The supernatant resulting from centrifuging the MAM fraction was further subjected to 100,000*g* for 1 hour, to pellet the pure MAMs. The supernatant containing microsomal (SR/ER) and cytosolic proteins was centrifuged at 20,000*g* for 30 minutes to remove mitochondrial and lysosomal contamination. The supernatant was further centrifuged at 100,000*g* for 1 hour. The resulting pellet contains an SR/ER fraction, and the supernatant is considered a cytosolic fraction.

### Seahorse measurements.

Mitochondria were isolated as described in the previous section and subjected to complex II–driven mitochondrial respiration using Seahorse XFe24 Analyzer (55, 56). Briefly, the mitochondrial pellet was resuspended in mitochondrial assay buffer 1 (MAS1; 220 mM d-mannitol, 70 mM sucrose, 10 mM KH_2_PO_4_, 5 mM MgCl_2_, 2 mM HEPES, 1 mM EGTA, and 0.2% [w/v] of fatty acid–free BSA, pH 7.4, adjusted with KOH). The protein concentration was estimated using a Bradford assay kit (Bio-Rad). After estimation, the mitochondria were pelleted again and resuspended in mitochondrial assay buffer 2 (MAS1 containing 10 mM succinate and 2 μM rotenone). About 5 μg of mitochondria in 100 μL of MAS2 was added to each well of the Seahorse XFe 24 plate and centrifuged at 2,000*g* for 20 minutes at 4°C. After verifying proper adherence of mitochondria, 400 μL of prewarmed (37°C) MAS1 with 5 mM ADP was added to each well, and the state III mitochondrial respiration was measured. Mitochondria were then treated with oligomycin (3.2 μM), FCCP (4 μM), and antimycin (4 μM). Seahorse protocol was followed; however, after each injection, 2 measurements were made to determine the OCR, the extracellular acidification rate, and various states of respiration (state III, state IV_0_, state IIIμ) (55). State III respiration represents the formation of ATP from ADP and inorganic phosphate, state IV_0_ respiration represents the proton leak because of the inhibition of the ATP synthase by oligomycin, and state IIIμ respiration represents the status of maximal respiratory capacity after FCCP treatment. Further, the RCR, an index of mitochondrial coupling, was calculated by dividing the values of state III/state IV_0_ (55, 56).

### Western blot analysis.

Total protein extraction and Western blotting were carried out as described before (17). Briefly, after protein transfer, the nitrocellulose membranes were stained with Ponceau S and cut into strips based on the molecular weight of each protein studied. The membrane strips were then blocked with 3% milk in PBS and probed overnight at 4°C using antibodies specific for SLN (anti-rabbit, 1:3,000, custom made) (57), PLN (anti-rabbit, 1:5,000, custom made) (57), RyR2 (anti-rabbit, 1:1,000, Thermo Fisher Scientific, PA5-77717), SERCA2a (anti-rabbit, 1:5,000, custom made) (57), MCU (anti-rabbit, 1:1,000, Cell Signaling Technology [CST], 149973), mitochondrial Ca^2+^ uptake 1 (MICU1; anti-rabbit, 1:1,000, CST, 12524), NCLX (anti-rabbit, 1:1,000, Thermo Fisher Scientific, PA5-114330), LETM1 (anti-rabbit, 1:1,000, ABclonal, A15685), TRPC1 (anti-rabbit, 1:1,000, Abcam, ab192031), total oxidative phosphorylation subunits cocktail (OXPHOS subunits; anti-mouse, 1:1,000, Abcam, ab110413), 4-HNE (anti-rabbit, 1:3,000, Abcam, ab46545), SOD2 (anti-mouse, 1:1,000, Santa Cruz Biotechnology, sc-137254), long-chain fatty-acid-coenzyme A ligase 4 (FACL4/ACSL4, anti-mouse, 1:1,000, Santa Cruz Biotechnology, sc-365230), glucose-regulated protein 75 (Grp75, anti-rabbit, 1:1,000, CST, 3593), α-tubulin (anti-mouse, 1:5,000, MilliporeSigma, T6199), lamin A/C (4C11, anti-mouse, 1:1,000, CST, 4777S), and cytochrome *c* oxidase IV (COX IV; anti-rabbit, 1:1,000, CST, 4850T). Membranes were incubated with appropriate secondary antibodies (anti-mouse, peroxidase-labeled, 0.2 μg/mL, material no. 5220-0342; and anti-rabbit, peroxidase-labeled, 0.2 μg/mL, material no. 5220-0336; Seracare) for 1 hour at room temperature and visualized with a SuperSignal West Dura Substrate kit (Thermo Fisher Scientific) using Bio-Rad ChemiDoc MP Imaging System. Quantitation of signals was performed using Image Lab version 5.1 software (Bio-Rad) and normalized to Ponceau S staining.

To detect the oxidative modification of proteins, total or mitochondrial protein fractions from the ventricles were derivatized using an OxyBlot protein oxidation detection kit (catalog S7150, MilliporeSigma), separated on 12% SDS-PAGE, and transferred to nitrocellulose membrane. The stable dinitrophenyl (DNP) proteins were detected by immunoblot using anti-DNP antibodies. Quantitation of signals was performed using Image Lab v. 5.1 software and normalized to Ponceau S staining.

### Complex activity assays.

The mitochondrial complex I (NADH dehydrogenase) and complex IV (cytochrome *c* oxidase) activities were measured in freshly prepared ventricular tissue lysates using Abcam assay kits (complex I, No. ab109721; complex IV, No. ab109911) as per the manufacturer’s instructions. For complex I activity, the optical density was measured at 450 nm in kinetic mode at room temperature (RT) for 30 minutes, and for complex IV activity, the optical density was measured at 550 nm in kinetic mode at RT for 120 minutes.

### Mitochondrial copy number.

Mitochondrial DNA copy number was calculated as the ratio of mitochondrial genome to nuclear genome following real-time qPCR using total DNA as described before (19, 58). Briefly, total DNA was prepared from the ventricular tissues using the Monarch Genomic DNA purification kit (New England Biolabs; T3010). The qPCR was performed using primers specific for mouse mitochondrial DNA (forward 5′-CTAGAAACCCCGAAACCAAA-3′; reverse, 5′-CCAGCTATCACCAAGCTCGT-3′) and nuclear DNA (beta 2-microglobulin; forward, 5′-ATGGGAAGCCGAACATACTG-3′; reverse 5′- CAGTCTCAGTGGGGGTGAAT-3′).

### Electron microscopy.

TEM was performed as described previously (59). Briefly, ventricular tissues were fixed overnight in Karnovsky’s solution (2% paraformaldehyde/2.5% glutaraldehyde in 0.1 M phosphate buffer). The fixed samples were then processed at the Rutgers Core Imaging Lab at Robert Wood Johnson Medical School. After postfixation with 1% osmium tetroxide in PBS for 1 hour, the tissue was dehydrated with a graded series of acetone concentrations and embedded in Spar resin. Sections of 98 nm thickness were placed on copper grids that were double-stained with uranyl acetate and lead citrate and examined under JEOL 1200 electron microscope. Images were captured and analyzed using NIH ImageJ software.

### Proteomics and data analysis.

Purified MAMs were resolved on 4%–20% Bio-Rad Mini-PROTEAN TGX precast gradient gels and subjected to LC-MS/MS on a Thermo Fisher Scientific Orbitrap Fusion Lumos Tribrid mass spectrometer at Rutgers Center for Advanced Proteomics Research facility. Three replicate experiments were performed in 2 batches. The resulting MS/MS spectra were searched against a SwissProt mouse database using the Sequest search engine on the Proteome Discoverer (V2.4) platform using a protein false discovery rate of less than 1%. The ratios between *mdx:utrn^–/–^* and WT and between *mdx:utrn^–/–^:sln^+/–^* and WT were calculated using the spectra counting method. In addition to ratio calculations, Partek Genomics Suite 7.0 was used for batch correction of raw spectral counts, followed by quantile normalization, pairwise comparisons using 1-way ANOVA, hierarchical clustering, and principal component analysis. Those proteins with a significant spectral count ratio > 2, multiple-hypothesis-corrected *P* < 0.05, and fold-difference > 1.5 were considered as most significantly different. GO Term Mapper was used to determine the subcellular localization of the various proteins identified, and IPA Software (QIAGEN) was used for functional analysis, including enriched biological processes, pathways, and upstream regulators.

### Statistics.

The data were analyzed in an unbiased and blinded manner to ensure that these results are consistent and reproducible. Each Western blotting experiment was repeated 2 or 3 times, and the mean values of these repeated experiments were used for quantitation. All statistical analyses were performed using GraphPad Prism v6.01 software. Ordinary 1-way ANOVA was used for multigroup comparison. Data were presented as mean ± SE. *P* < 0.05 was considered significant.

### Study approval.

All animal procedures were approved by the Institutional Animal Care and Use Committee of NJMS, Rutgers, according to the *Guide for the Care and Use of Laboratory Animals* published by the US National Academies Press (NIH, 8th edition, 2011).

### Data availability.

All data presented in this article are included in the main text, supplemental figures, and Supporting Data Values file.

## Author contributions

GJB and SM conceived and designed research; SM, NF, CLG, GP, and RM analyzed data; SM, LHX, CLG, JS, and GJB interpreted results; SM and GJB drafted the manuscript; SM, LHX, CLG, JS, and GJB edited the manuscript; and SM, NF, CLG, GP, RM, JS, LHX, and GJB approved the final version of the manuscript.

## Supplementary Material

Supplemental data

Unedited blot and gel images

Supporting data values

## Version 1 04/02/2024

In-Press Preview

## Version 2 05/08/2024

Electronic publication
