# Supplementary material for: Improved mitochondrial function in the hearts of sarcolipin-deficient dystrophin and utrophin double-knockout mice
Source: JCI Insight. 2024 Apr 2;9(9):e170185. doi: 10.1172/jci.insight.170185 (PMC11141945; doi:10.1172/jci.insight.170185)
Supplement: Supplemental data [file jciinsight-9-170185-s015.pdf]

Supplementary Table For

Improved mitochondrial function in the heart of sarcolipin-deficient dystrophin and utrophin double knockout mice

Satvik Mareedu[1], Nadezhda Fefelova[1], Cristi L. Galindo[2], Goutham Prakash[1], Risa Mukai[1], Junichi Sadoshima[1], Lai-Hua Xie[1], Gopal J Babu[1*]

[1]Department of Cell Biology and Molecular Medicine, New Jersey Medical School, Rutgers University, Newark, NJ 07103, [2]Vascular Medicine Institute & Cardiology, University of Pittsburgh, Pittsburgh, PA 15213.

*Corresponding author
Gopal J Babu, PhD
Department of Cell Biology and Molecular Medicine
Rutgers, New Jersey Medical School
185 South Orange Ave, Newark, NJ 07103
p. 973-972-5376 f. 973-972-7489
Email: babugo@njms.rutgers.edu

Supplementary Table 1: Baseline echocardiographic data of 3 months old mice

	WT (n=6)	*mdx:utrn*$^{-/-}$ (n=5)	*mdx:utrn*$^{-/-}$: *sln*$^{+/-}$ (n=6)
**IVSd, mm**	0.62±0.01	0.55±0.01$^{\$}$	0.58±0.03
**IVSs, mm**	0.77±0.02	0.67±0.02$^{\$**}$	0.86±0.02
**LVIDd, mm**	4.02±0.03	3.83±0.11	3.88±0.05
**LVIDs, mm**	2.46±0.03	2.72±0.11$^{\$*}$	2.42±0.04$^{\$}$
**LVPWd, mm**	0.67±0.02	0.59±0.01$^{\$}$	0.64±0.02
**LVPWs, mm**	0.9±0.01	0.67±0.02$^{\$\$**}$	0.93±0.03
**FS (%)**	39±0.3	29±1.2$^{\$\$**}$	38±0.4$^{\$}$
**EF (%)**	70±0.4	56±2.0$^{\$\$**}$	69±0.5$^{\$}$
**HR, bpm**	400±4	433±22*	361±14

IVSd- interventricular septal end diastole, IVSs- interventricular septal end systole, LVIDd- left ventricular internal diameter end diastole, LVIDs- left ventricular internal diameter end systole, LVPWd- left ventricular posterior wall end diastole, LVPWs- left ventricular posterior wall end systole, FS- fractional shortening, EF-ejection fraction, HR-heart rate. $^{\$}p<0.05$ vs. WT; $^{\$\$}p<0.0001$ vs. WT; *p<0.05 vs. other groups; **p<0.0001 vs. other groups.

Supplementary Figures For

Improved mitochondrial function in the heart of sarcolipin-deficient dystrophin and utrophin double knockout mice

Satvik Mareedu[1], Nadezhda Fefelova[1], Cristi L. Galindo[2], Goutham Prakash[1], Risa Mukai[1], Junichi Sadoshima[1], Lai-Hua Xie[1], Gopal J Babu[1*]

[1]Department of Cell Biology and Molecular Medicine, New Jersey Medical School, Rutgers University, Newark, NJ 07103, [2]Vascular Medicine Institute & Cardiology, University of Pittsburgh, Pittsburgh, PA 15213.

*Corresponding author
Gopal J Babu, PhD
Department of Cell Biology and Molecular Medicine
Rutgers, New Jersey Medical School
185 South Orange Ave, Newark, NJ 07103
p. 973-972-5376 f. 973-972-7489
Email: babugo@njms.rutgers.edu

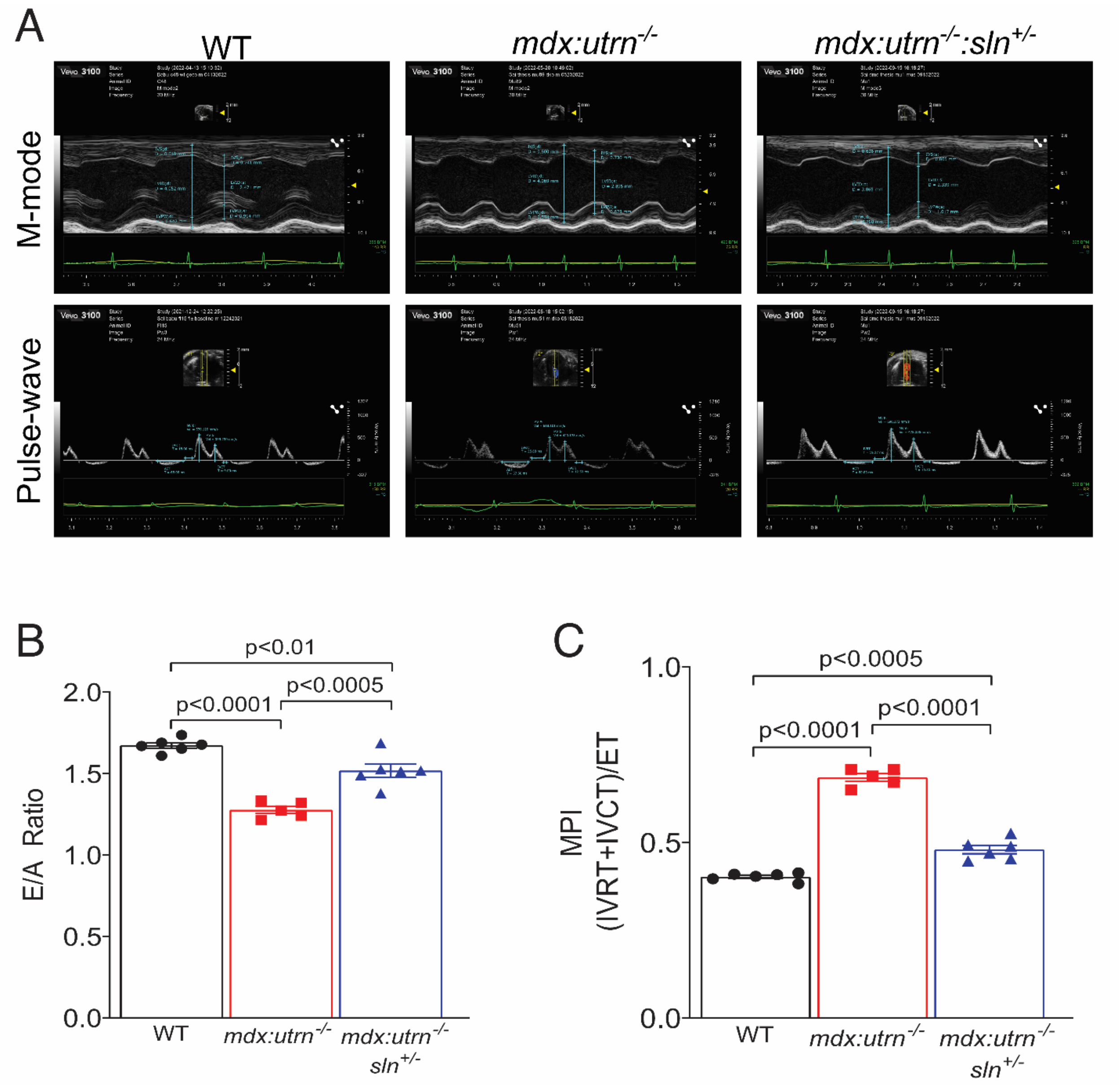

Supplementary Figure 1: Reducing SLN expression prevents diastolic dysfunction and improves overall cardiac function in *mdx:utrn*$^{-/-}$ mice. (A) Representative M-mode and pulse-wave Doppler imaging of apical 4-chamber view of hearts from WT, *mdx:utrn*$^{-/-}$, and *mdx:utrn*$^{-/-}$*:sln*$^{+/-}$ mice. **(B)** E/A ratio and **(C)** MPI, which is calculated following the sum of pulse-wave derived isovolumetric relaxation time (IVRT), and isovolumetric contraction time (IVCT) divided by ejection time (ET). n=6 for WT and *mdx:utrn*$^{-/-}$*:sln*$^{+/-}$ mice & n=5 for *mdx:utrn*$^{-/-}$ mice. Data were analyzed by Ordinary one-way ANOVA for multigroup comparisons. Values shown are means ±SE.

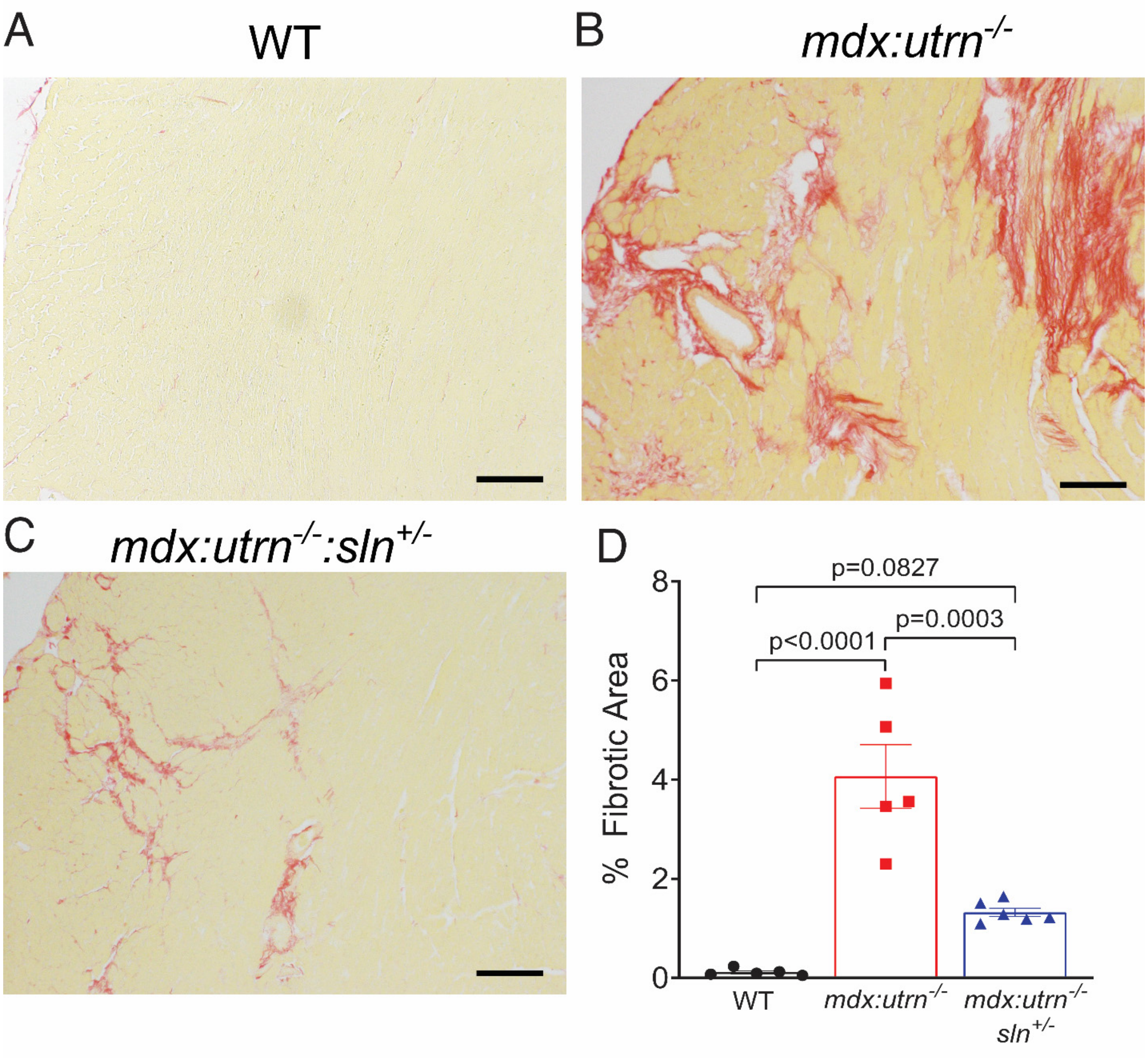

Supplementary Figure 2: Fibrosis is reduced in the ventricles of *mdx:utrn$^{-/-}$:sln$^{+/-}$* mice. Representative images showing PSR stained ventricular sections from **(A)** WT, **(B)** *mdx:utrn$^{-/-}$* and **(C)** *mdx:utrn$^{-/-}$:sln$^{+/-}$* mice. The original magnification is 10x. Scale bar =100 µm. **(D)** Quantitation showing fibrotic areas calculated from the red PSR-stained collagen fibers. n=5 for WT and *mdx:utrn$^{-/-}$* mice; n=6 for *mdx:utrn$^{-/-}$:sln$^{+/-}$* mice. Data were analyzed by Ordinary one-way ANOVA for multigroup comparisons. ns-not statistically significant. Values shown are means ±SE.

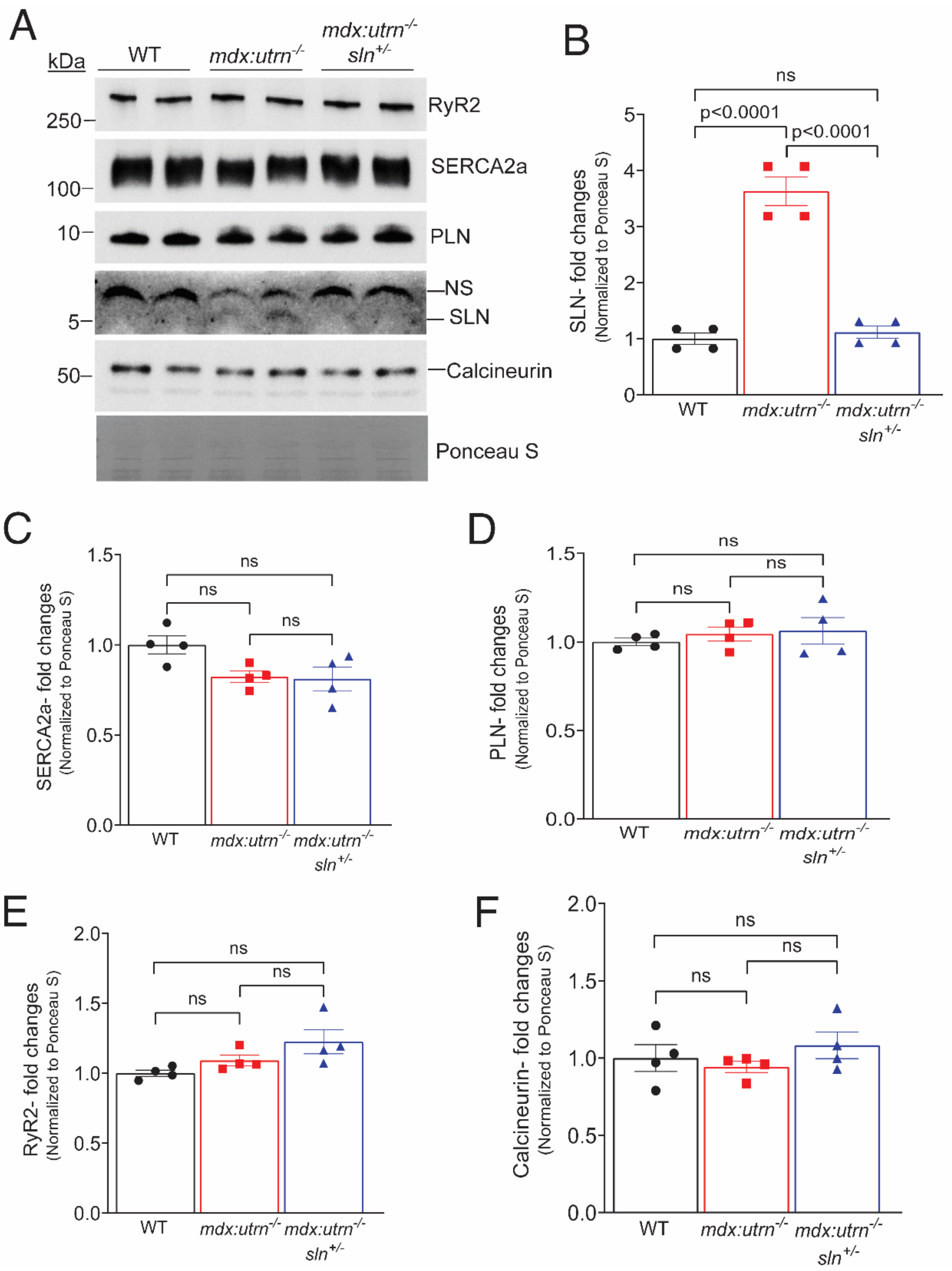

Supplementary Figure 3: Sarcolipin reduction does not affect the expression of major SR Ca^{2+} handling proteins in the ventricles of *mdx:utrn*$^{-/-}$ mice. (A) Representative western blots and quantitation showing **(B)** SLN, **(C)** SERCA2a, **(D)** PLN, **(E)** RyR2, and **(F)** calcineurin protein levels in the ventricles of WT, *mdx:utrn*$^{-/-}$ and *mdx:utrn*$^{-/-}$*:sln*$^{+/-}$ mice. n=4 mice per genotype. For western blotting, 10-40 µg of protein is loaded per well. Data were analyzed by Ordinary one-way ANOVA for multigroup comparisons. ns-not statistically significant. Values shown are means ±SE.

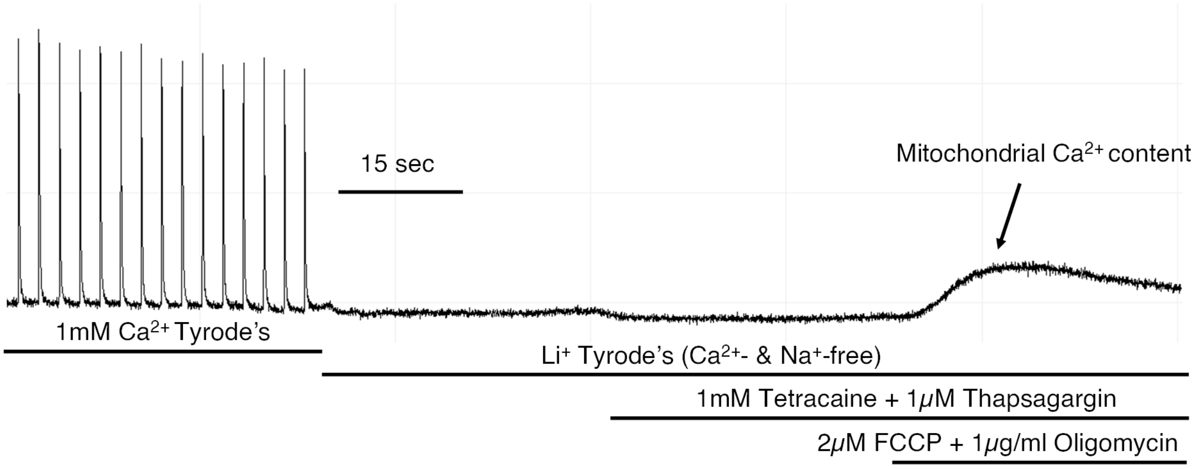

Supplementary Figure 4: A representative Ca^{2+} trace showing twitch Ca^{2+} transients elicited by field stimulation at 0.5 Hz, followed by no stimulation and treating the myocytes with Li^+ Tyrode's free of Na^+ and Ca^{2+}, followed by inhibition of SR Ca^{2+} release with 1mM tetracaine and 1μM thapsigargin and stimulating mitochondrial Ca^{2+} efflux/transient (mitochondrial Ca^{2+} content) by treating with 2μM FCCP and 1μg/ml oligomycin.

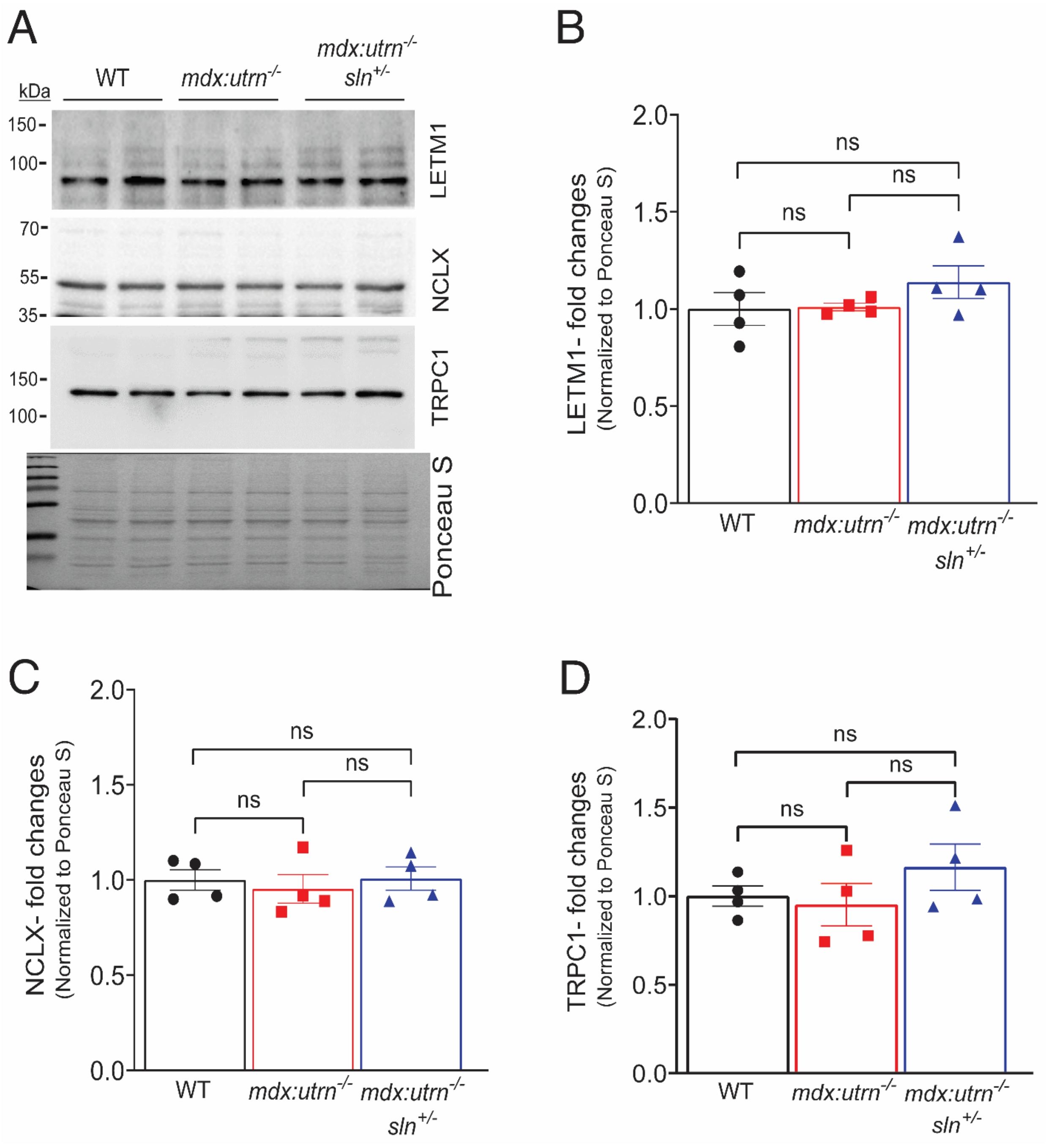

Supplementary Figure 5: Mitochondrial Ca^{2+} handling proteins are unaltered in the ventricles of WT, *mdx:utrn$^{-/-}$* and *mdx:utrn$^{-/-}$:sln$^{+/-}$* mice. (A) Representative western blots and quantitation showing **(B)** LETM1, **(C)** NCLX, and **(D)** TRPC1 protein levels in the ventricles of WT, *mdx:utrn$^{-/-}$* and *mdx:utrn$^{-/-}$:sln$^{+/-}$* mice. For western blotting, 10 µg of protein is loaded per well. n=4 mice per genotype. Data were analyzed by Ordinary one-way ANOVA for multigroup comparisons. ns-not statistically significant. Values shown are means ±SE.

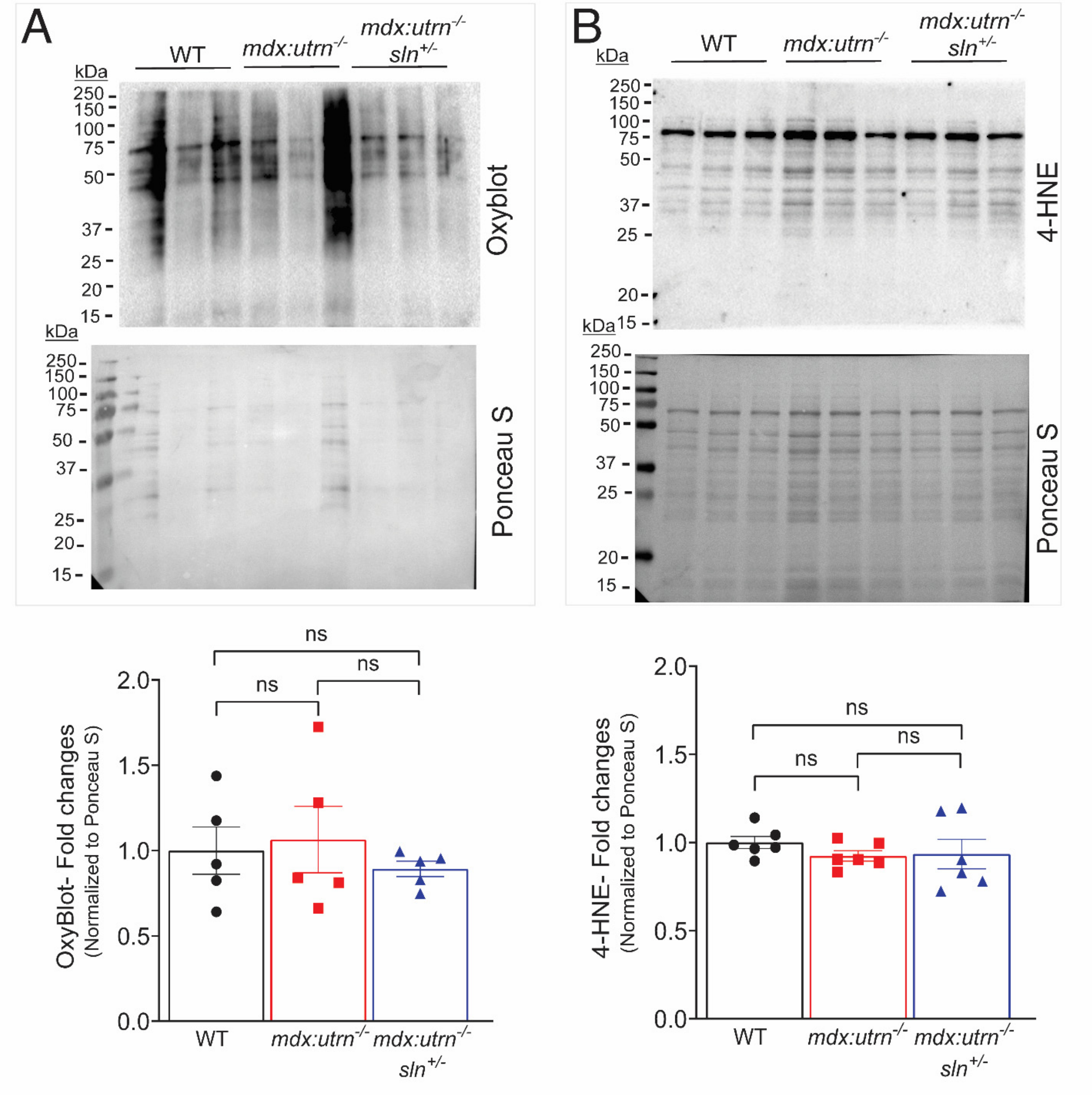

Supplementary Figure 6: Protein carbonylation and lipid peroxidation are unaltered in the heart mitochondria purified from WT, *mdx:utrn$^{-/-}$* and *mdx:utrn$^{-/-}$:sln$^{+/-}$* mice. Representative **(A)** OxyBlot and **(B)** 4-HNE western blots (top) and quantitation (bottom) showing the levels of carbonylated proteins and lipid peroxidation in the heart mitochondrial proteins prepared from WT, *mdx:utrn$^{-/-}$* and *mdx:utrn$^{-/-}$:sln$^{+/-}$* mice. For western blotting, 10 µg of protein is loaded per well. n=5 for Oxyblot and n=6 for 4-HNE. Data were analyzed by Ordinary one-way ANOVA for multigroup comparisons. ns-not statistically significant. Values shown are means ±SE.

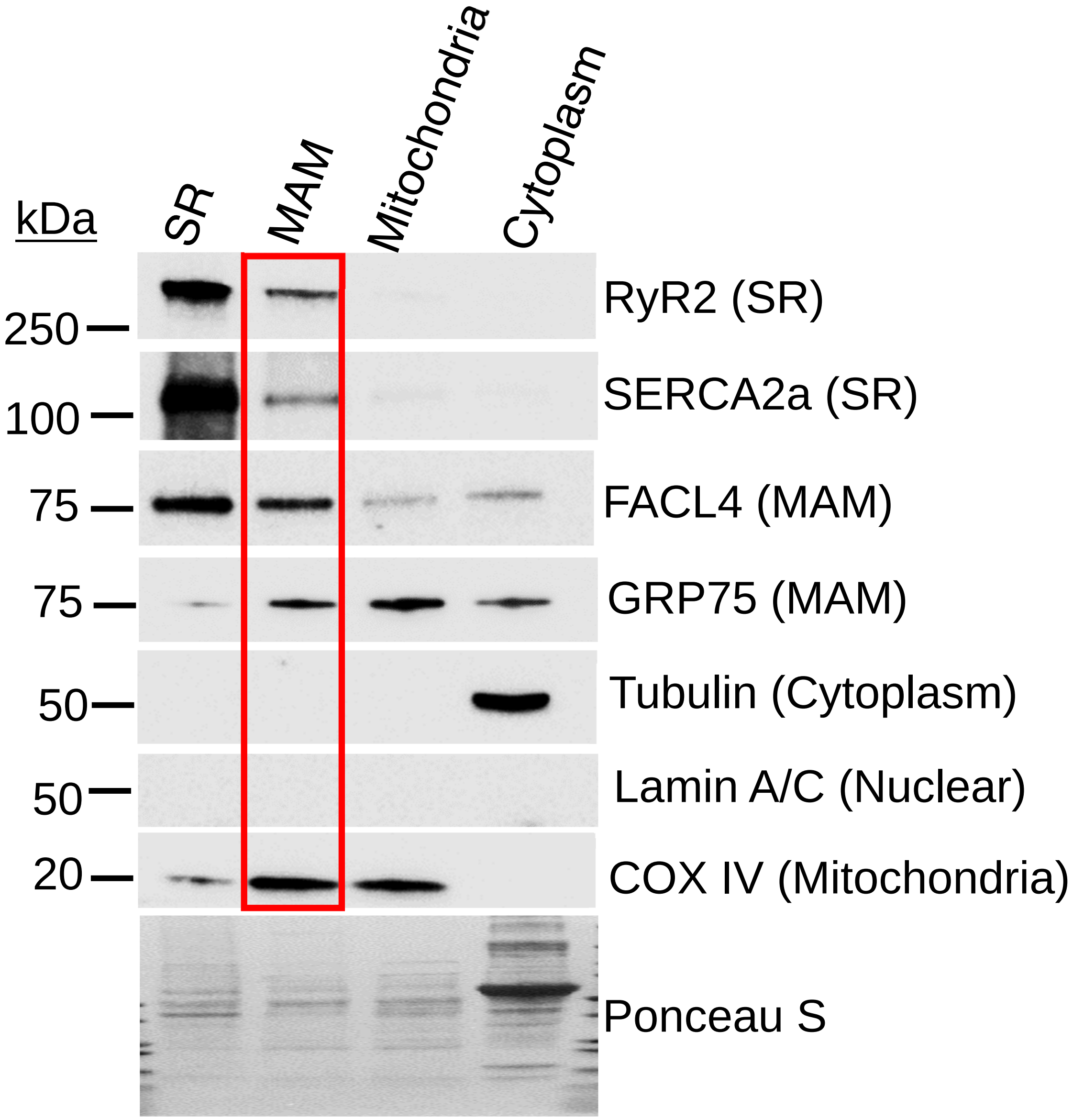

Supplementary Figure 7: Purity of MAMs preparation. Representative western blots showing the purity of MAMs prepared from ventricles of wild type mice. For western blotting, 10 μg of protein is loaded per well. SR-sarcoplasmic reticulum; RyR2-ryanodine receptor 2; SERCA2a-sarco/endoplasmic reticulum Ca^{2+} ATPase; FACL4-long-chain fatty-acid-coenzyme A ligase 4; GRP75- glucose-regulated protein 75; COX IV- cytochrome c oxidase IV.

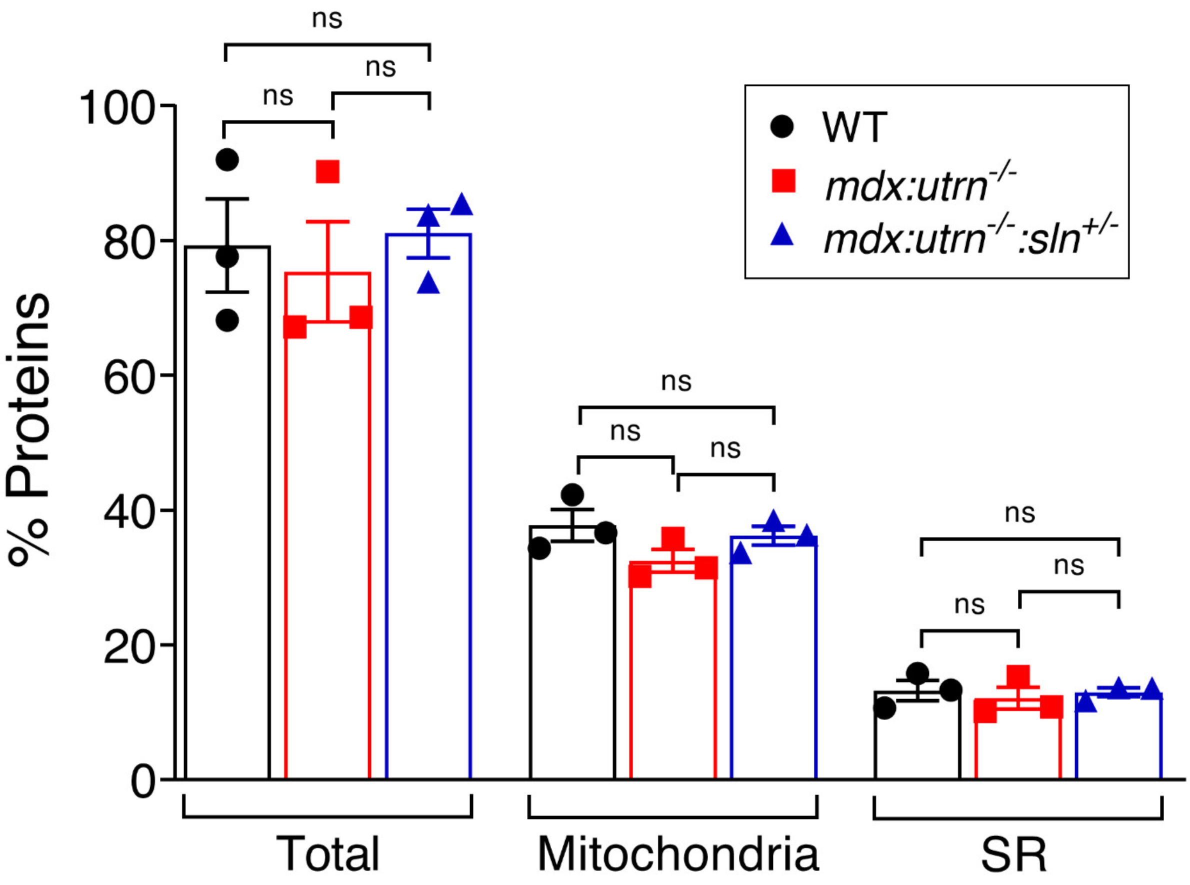

Supplementary Figure 8: Proteomic analysis of MAMs. The GO Term Mapper analysis shows the number of total proteins, and mitochondria & SR component proteins (in percentage) in the MAMs prepared from WT, *mdx:utrn*$^{-/-}$ and *mdx:utrn*$^{-/-}$*:sln*$^{+/-}$ ventricles. 4-5 ventricles were pooled for each MAM preparation. n=3 MAMs preparation/per genotype. Data were analyzed by Ordinary one-way ANOVA for multigroup comparisons. ns-not statistically significant. Values shown are means ±SE.
