# Supplementary figures and images for: Improved mitochondrial function in the hearts of sarcolipin-deficient dystrophin and utrophin double-knockout mice
Source: JCI Insight. 2024 Apr 2;9(9):e170185. doi: 10.1172/jci.insight.170185 (PMC11141945; doi:10.1172/jci.insight.170185)

Figure 2C

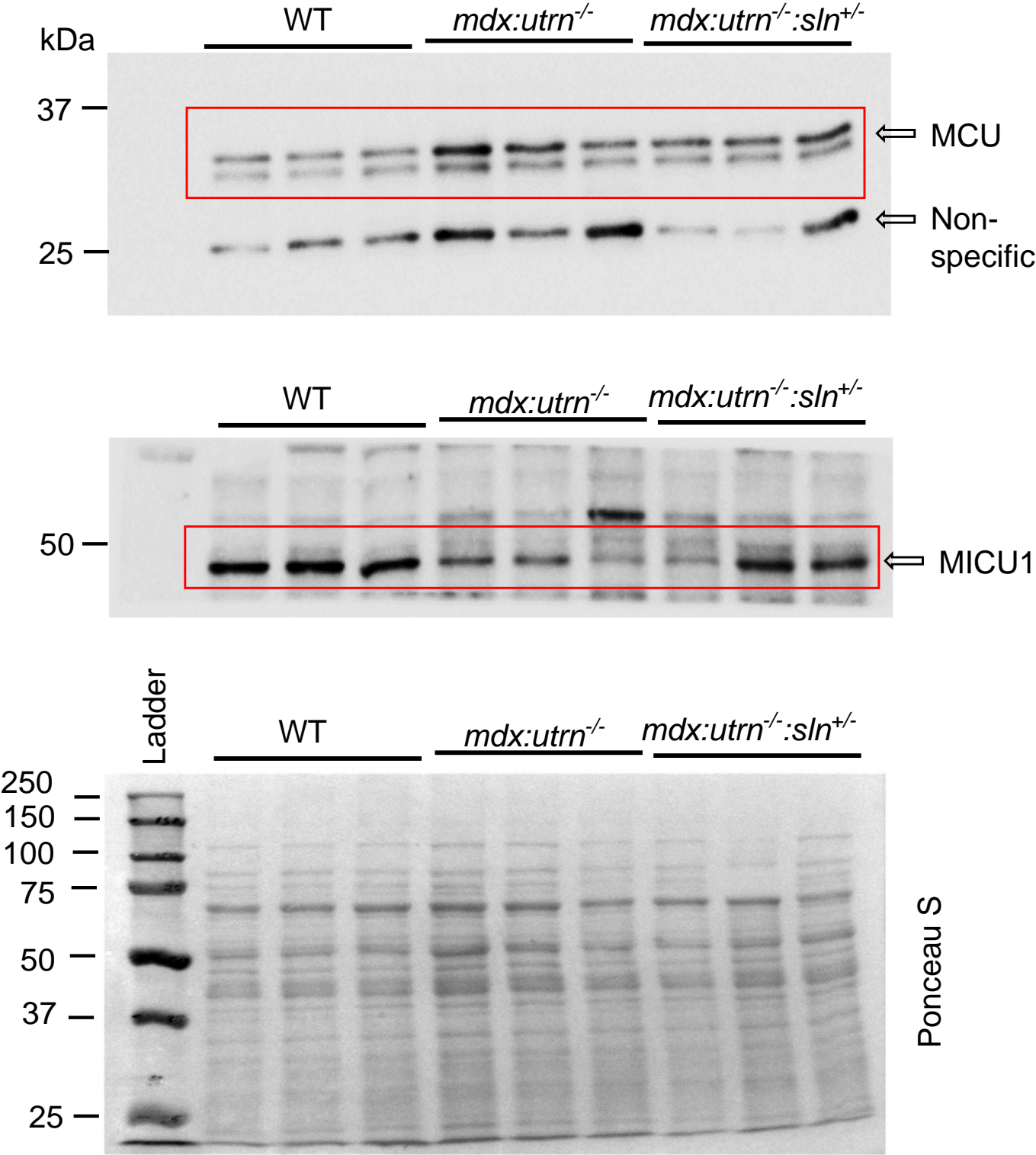

Figure 3A

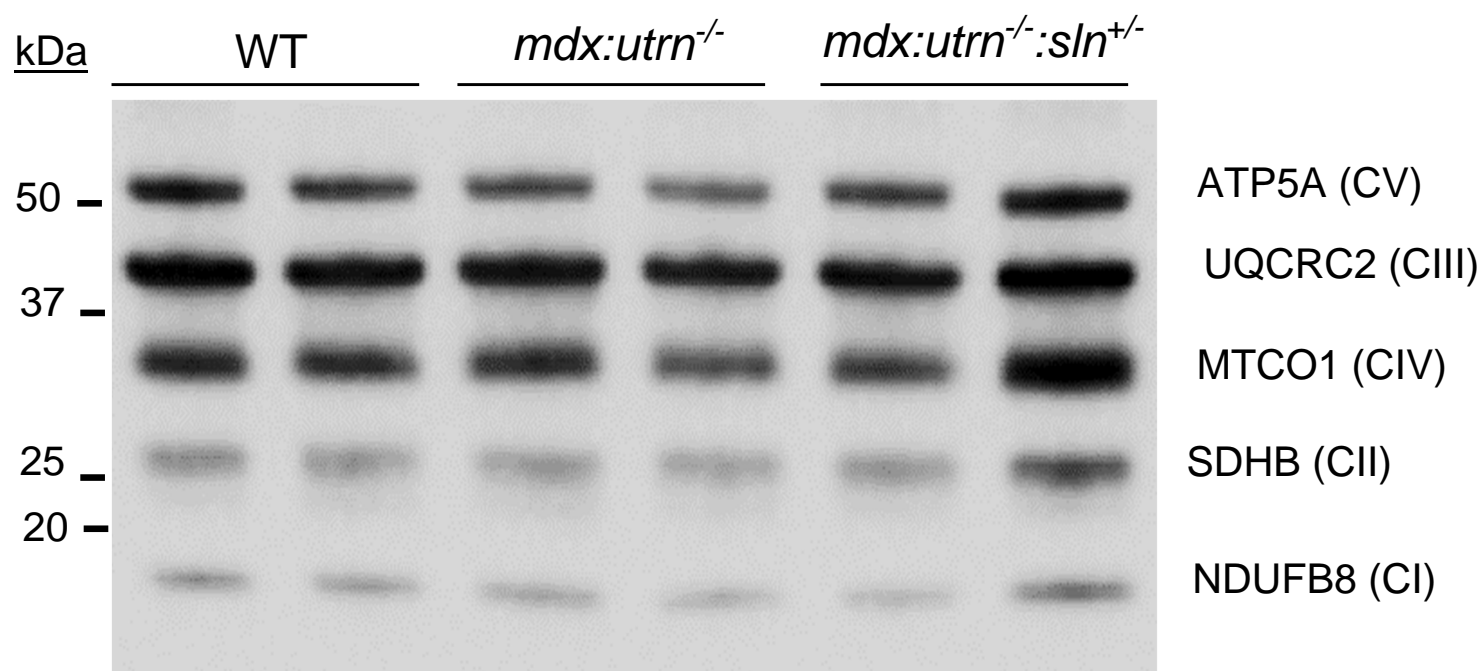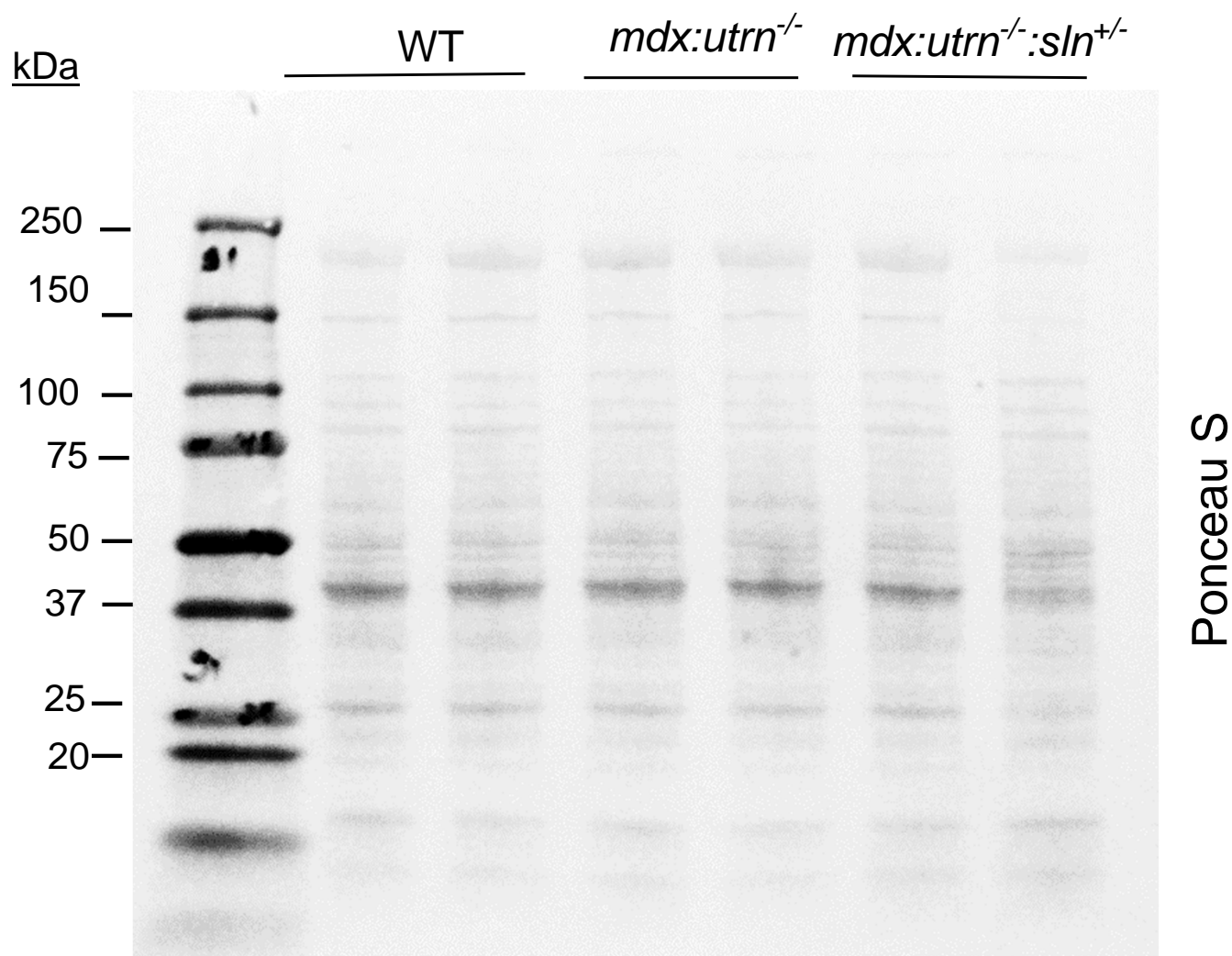

Figure 4E

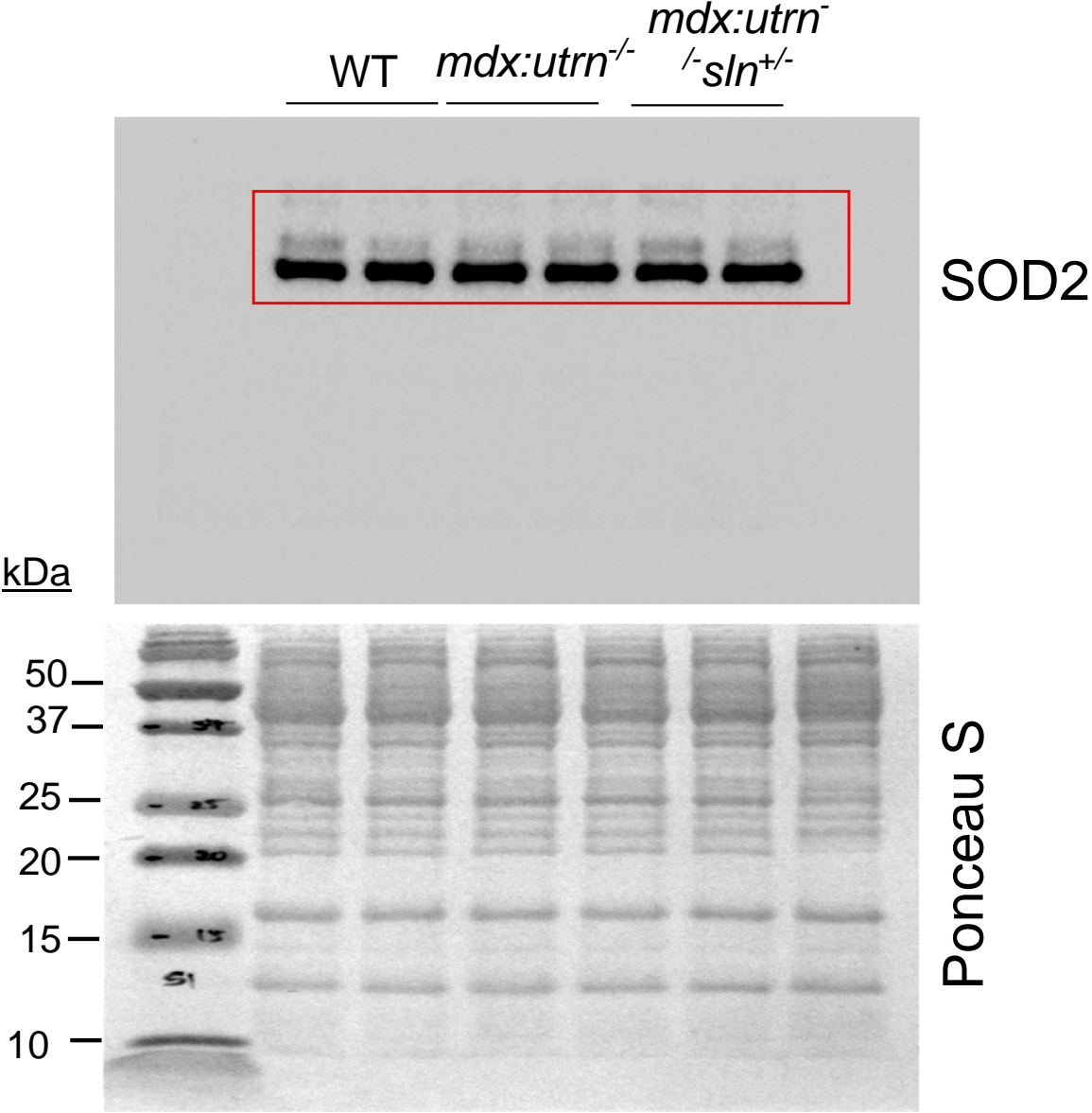

Figure 6B

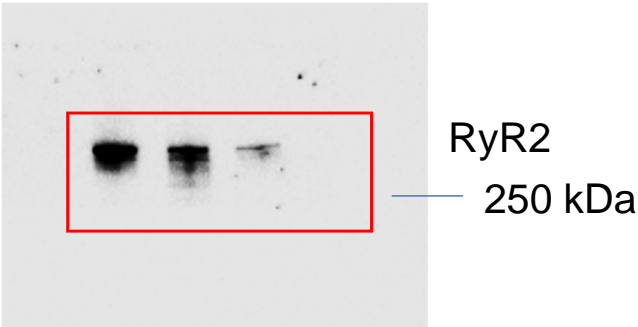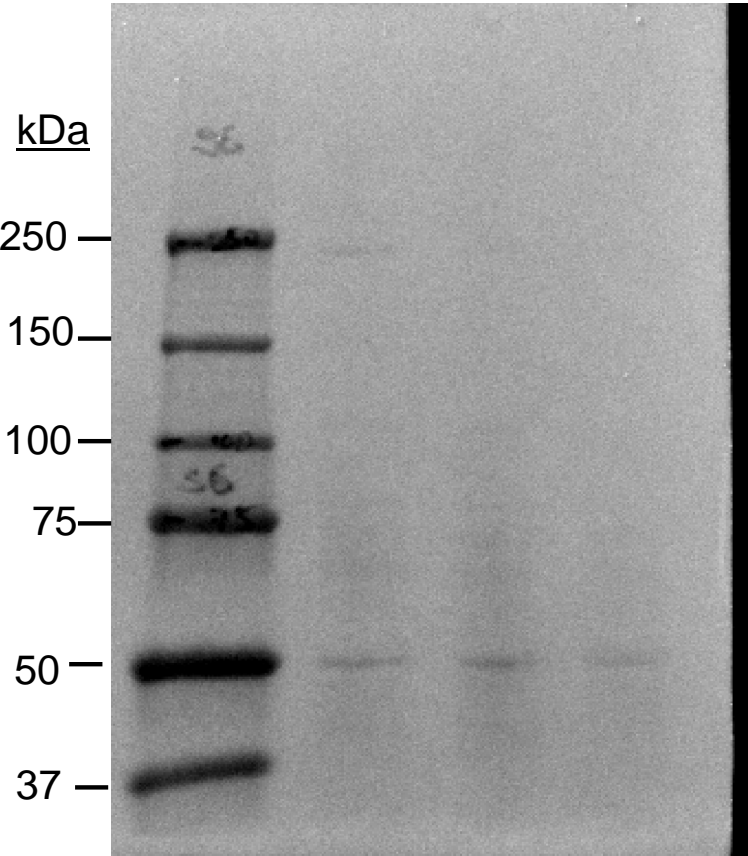

Figure 6B

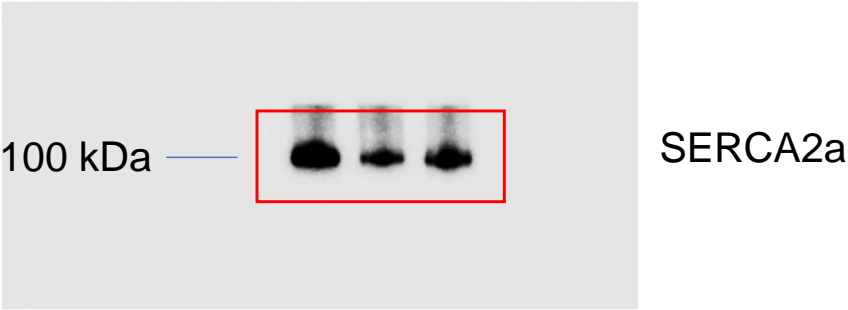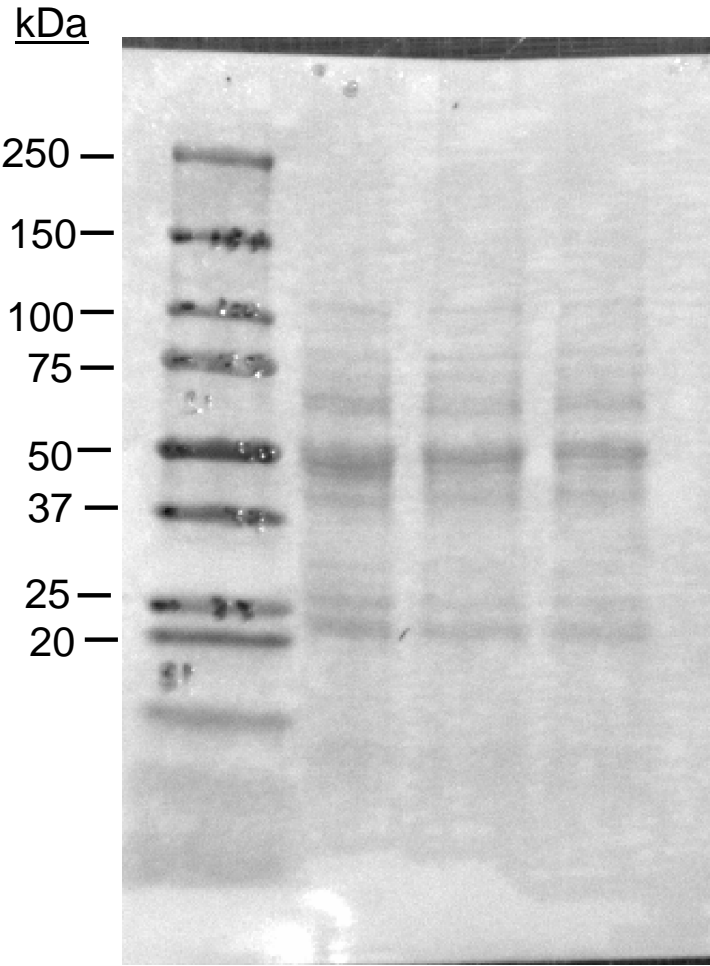

Figure 6B

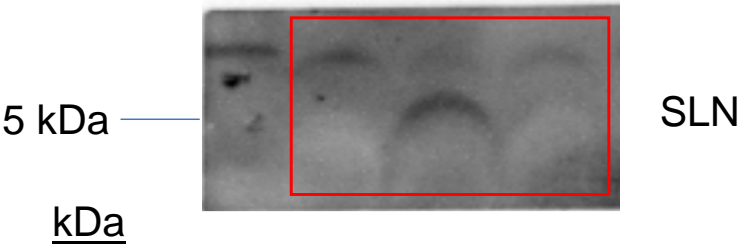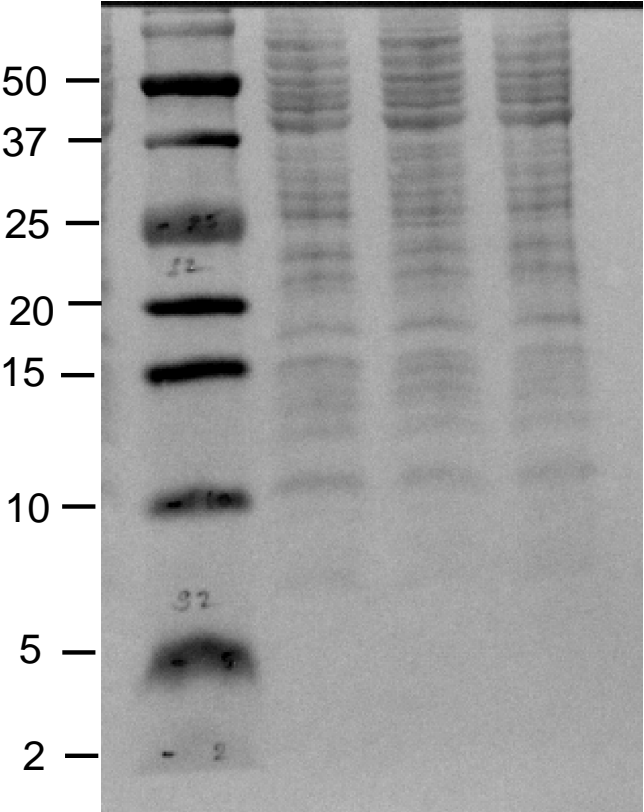

Figure 6B

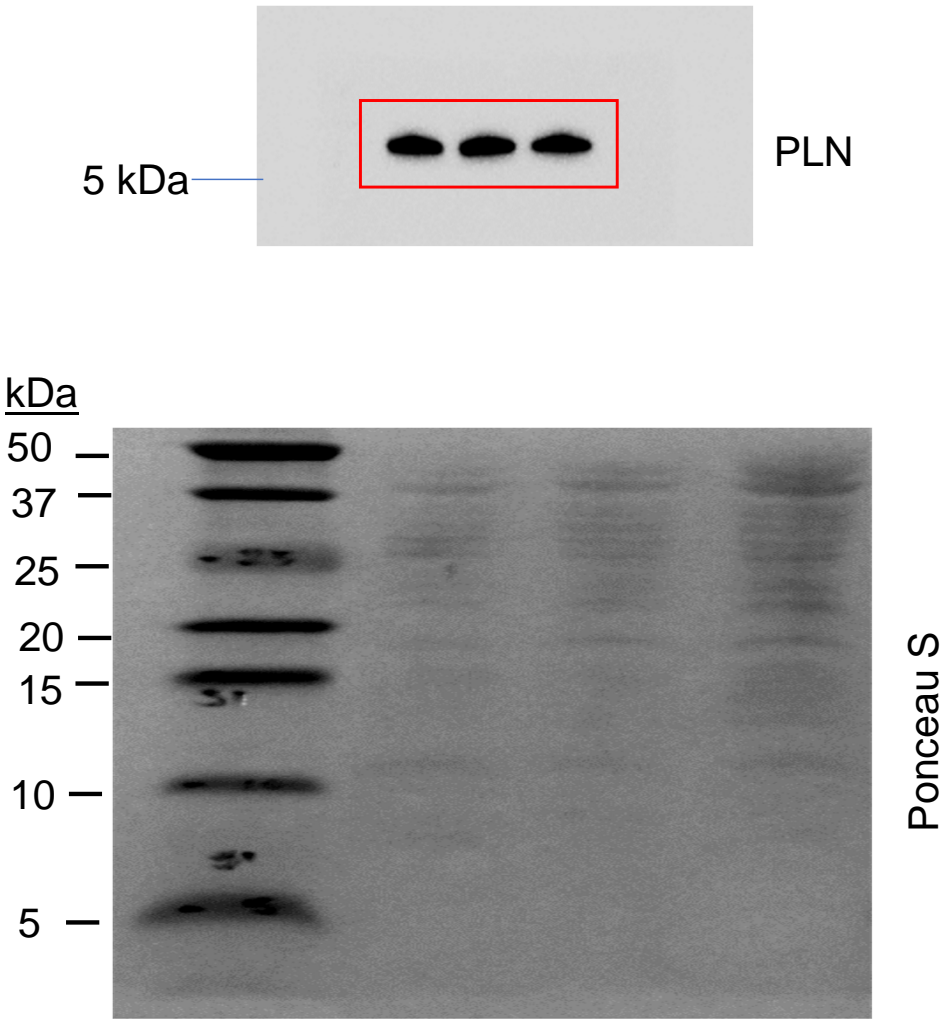

Supplement: Unedited blot and gel images [file jciinsight-9-170185-s016.pdf]
